# Effect of Hot-Air Drying Temperature on the Physicochemical, Functional, Compositional, and Structural Properties of Catfish (*Clarias macrocephalus* × *C. gariepinus*) Powder

**DOI:** 10.3390/foods15142443

**Published:** 2026-07-09

**Authors:** Narin Charoenphun, Zhaojun Ban, Paramee Noonim, Somwang Lekjing, Thanamat Paongoen, Karthikeyan Venkatachalam

**Affiliations:** 1 Faculty of Science and Arts, Burapha University, Chanthaburi Campus, Chanthaburi 22170, Thailand; narinch@buu.ac.th; 2 School of Biological and Chemical Engineering, Zhejiang University of Science and Technology, Hangzhou 310023, China; banzhaojun@zust.edu.cn; 3 Faculty of Innovative Agriculture, Fisheries and Food, Prince of Songkla University, Surat Thani Campus, Makham Tia, Mueang, Surat Thani 84000, Thailand; paramee.n@psu.ac.th (P.N.); somwang.s@psu.ac.th (S.L.); 6840620202@psu.ac.th (T.P.)

**Keywords:** catfish powder, hot-air drying, tray drying, proximate composition, fatty acid profile, functional properties, lipid oxidation, food fortification

## Abstract

Catfish (*Clarias macrocephalus* × *C. gariepinus*) is economically important but perishable, and hot-air drying yields shelf-stable catfish powder (CFP); however, its quality response to high-temperature drying (>100 °C) remains poorly understood. The present study aimed to examine the effects of five drying temperatures (60–180 °C) on the physicochemical, nutritional, functional, structural, volatile, oxidative, and microbiological properties of CFP. Drying exerted parameter-specific effects. Moisture and water activity fell from 6.24% to 4.75% and 0.42 to 0.23, with microbial counts reduced at ≥120 °C. Browning raised lightness, redness, and yellowness from 49.53 to 57.64, 1.94 to 2.97, and 13.95 to 18.14, respectively. Protein concentrated from 57.63% to 62.24%, whereas oxidation lowered fat from 22.49% to 17.17%. Minerals rose marginally, although iron and zinc declined. Amino acids were largely preserved, though methionine (2.71 to 2.21 g/100 g protein) and cysteine declined and glycine and proline increased. DHA declined most, from 4.61% to 3.20%, followed by EPA and AA. Water holding, oil holding, wettability, dispersibility, and colloidal stability decreased, whereas emulsion stability decreased from 57.14% at 60 °C to 42.10% at 180 °C. Zeta potential became less negative (−35.82 to −20.74 mV), with larger particle size and polydispersity. Among 72 volatiles across 11 classes, 60–90 °C favored fishy aldehydes and 2-pentyl furan and ≥150 °C generated Maillard and sulfur compounds, whereas 120 °C produced the most balanced volatile profile, with lower relative abundance of fishy aldehydes than at 60–90 °C, consistent with the intermediate TBARS values and comparatively better retention of unsaturated fatty acids at this temperature, and limited accumulation of Maillard-derived and sulfur compounds relative to 150–180 °C. FTIR, XRD, TGA, and SEM confirmed preserved structure. Overall, the results showed that 120 °C provided the best balance of dehydration, nutrient and functional retention, flavor, and microbial safety.

## 1. Introduction

Global initiatives to improve food and nutritional security have increasingly focused on developing scalable and cost-effective post-harvest technologies, especially for nutrient-rich yet highly perishable aquatic commodities like catfish (*Clarias macrocephalus × C. gariepinus*) [1,2]. Global catfish aquaculture production approached 6.9 million metric tons in 2023, with air-breathing catfish (primarily *Clarias* species) accounting for approximately 1.84 million metric tons, representing 26.6% of total catfish production, and being cultivated in more than 60 countries worldwide [3]. In Southeast Asia, Indonesia leads in *Clarias* production (contributing approximately 22% of global catfish tonnage), while the commercial hybrid *Clarias macrocephalus* × *C. gariepinus* has attained economic importance in Thailand and neighboring countries owing to its rapid growth, disease tolerance, and desirable organoleptic characteristics [3]. In numerous low- and middle-income countries, catfish and other freshwater fish species serve as vital and accessible sources of dietary protein, significantly contributing to local food systems and nutritional adequacy [4]. Nutritionally, their flesh is notable for its high protein content, a complete range of essential amino acids, and substantial levels of lipid-associated nutrients, making them a crucial source of quality animal protein in diets often lacking diversity [5]. As summarized in Table S1, drying substantially concentrates crude protein in *C. gariepinus* from approximately 17–19% in fresh muscle to 52–73% on a wet weight basis in dried products, with a concomitant reduction in moisture to below 10%, underscoring the nutritional potential of hot-air-dried catfish powder as a protein-dense ingredient for food fortification and functional food applications [5,6,7,8].

However, these nutritional benefits, particularly the high moisture content and active post-mortem metabolism, make catfish highly prone to rapid spoilage. The particularly rapid spoilage of freshwater catfish relative to many marine species could possibly be attributed to several interrelated factors: the higher ambient temperature of tropical freshwater environments that supports faster growth of mesophilic spoilage bacteria; the comparatively elevated water activity of catfish flesh (a_w_~0.99); the high activity of endogenous proteases and lipases in *Clarias* species, which accelerate autolysis and lipolysis post-mortem; and the rich supply of free amino acids and non-protein nitrogen that serve as readily available substrates for microbial proliferation. Collectively, these characteristics render freshwater catfish more susceptible to quality deterioration than leaner marine fish or red meat under ambient storage conditions [9]. While conventional preservation methods, such as refrigeration and freezing, can effectively extend shelf life, they are often impractical in resource-limited settings due to their reliance on a continuous energy supply and robust cold chain infrastructure. In contrast, thermal dehydration, particularly hot air drying, has emerged as a promising alternative [10]. By reducing water activity, hot air drying inhibits microbial growth and transforms fresh catfish into a lightweight, shelf-stable product [6]. This dried material can be further processed into a powder, extending its usability and supporting product diversification through improved shelf life and transportability [11]. Such powders are well-suited for decentralized processing models and can serve as versatile ingredients in food fortification, flavor enhancement, and convenience food formulations [1].

Despite its potential, most existing research has concentrated on low to moderate drying temperatures (typically 40–80 °C). While these temperatures are gentle on heat-sensitive nutrients, they often lead to prolonged drying times, inadequate microbial inactivation, and increased cumulative energy consumption [7,11]. These limitations have sparked growing interest in high-temperature drying (90–180 °C) as a more time-efficient and scalable alternative, owing to its well-documented advantages in industrial food processing, including significantly reduced drying times, enhanced microbial inactivation, and improved control over key product attributes [12,13,14]. Exposure to elevated temperatures effectively inactivates vegetative bacteria through irreversible membrane damage and inhibition of essential enzymatic activity, thereby enhancing the microbiological safety of dried fish products and powders [11,14]. However, high-temperature drying can negatively impact powder reconstitution behavior, wettability, and flow-related properties, underscoring the importance of optimizing processing conditions to balance efficiency with product quality [12,15]. While high-temperature drying offers functional advantages, elevated thermal loads can introduce quality trade-offs, including protein denaturation, lipid oxidation, Maillard reaction browning, and structural alterations that compromise solubility, amino acid bioavailability, and the oxidative stability of lipid-associated components [13,16]. These degradative effects highlight the importance of tightly controlled processing conditions to preserve the nutritional and functional integrity of fish-based materials [11]. Nevertheless, thermally induced changes are not uniformly detrimental; drying temperature has been shown to influence proximate composition, lipid oxidation products, and fatty acid profiles in ways that reflect both degradative and transformative thermal effects [7,16]. In this context, hot air-drying functions not only as a preservation method but also as a deliberate processing technique that can be leveraged to modify and enhance product characteristics. This approach is particularly effective for developing fish powders intended for specialized applications, including high-protein nutritional supplements, natural flavoring components, and functional ingredients with relevance to health and culinary innovation [12,14].

Despite the growing interest in high-temperature drying technologies for fish processing, the physicochemical, nutritional, and structural changes that occur at temperatures exceeding 100 °C remain inadequately understood. Protein unfolding and oxidation are key mechanisms in this thermal range, with studies showing that increased heating time and temperature significantly enhance protein surface hydrophobicity, aggregation, and carbonyl formation while reducing thiol groups, which collectively alter solubility, emulsification, and gelation properties. Lipid oxidative stability deteriorates under severe heat exposure, leading to accumulation of primary and secondary oxidation products that intensify oxidative degradation pathways. In general, as the processing conditions of fish reach higher temperatures, the accumulation of primary and secondary lipid oxidation products becomes more pronounced, highlighting the intensification of oxidative degradation pathways under severe thermal exposure [16]. Excessive thermal exposure in these conditions can lead to degradation phenomena, such as nutrient depletion and irreversible structural damage to the protein and lipid matrix [11]. Meanwhile, the color characteristics of food powders are significantly affected by drying temperatures, with higher temperatures promoting browning through thermally driven reactions that alter the visual and sensory profile of the dried product [17]. This intersection of degradation risk and functional modification defines a critical area of research and technological innovation within fish product engineering. Fish powder is increasingly recognized as a multifunctional ingredient with diverse applications across the food industry [18]. Its high nutritional density, particularly in terms of protein and mineral content, including calcium and phosphorus from bone-rich fractions, makes it especially effective for food fortification strategies targeting nutrient-deficient populations [19]. When incorporated into cereals, soups, snacks, weaning foods, and ready-to-eat meals, fish powder enhances the overall nutritional profile without significantly altering sensory attributes, thus supporting its integration into culturally familiar diets [20]. The functional properties of fish protein powder, including water-holding, emulsification, and gelation, further support its incorporation into a variety of processed food systems as a structurally active ingredient [21]. In response to increasing consumer demand for high-protein, nutrient-rich, and minimally processed foods, fish powder emerges as a sustainable and adaptable ingredient that aligns with both health-focused product development and evolving culinary preferences [18]. Protein unfolding and oxidation are key mechanisms in this thermal range, with studies showing that increased heating time and temperature significantly enhance protein surface hydrophobicity, aggregation, and carbonyl formation while reducing thiol groups, which collectively alter solubility, emulsification, and gelation properties [17].

To the best of our knowledge, this research is among the first to systematically examine the effects of drying temperatures, reaching up to 180 °C, on the production of catfish powder (CFP) from *Clarias macrocephalus* × *Clarias gariepinus*. It addresses a significant gap in the current understanding of thermal degradation processes and functional transformations in aquatic proteins. Specifically, the study investigates how hot air drying within a temperature range of 60–180 °C affects the compositional, structural, and functional properties of catfish powder. The aim is to identify critical temperature thresholds that optimize product quality while enhancing the scalability of high-temperature drying for aquaculture-based food applications. By linking structural and functional modifications to practical outcomes in food processing and preservation, this work contributes to post-harvest innovation in fishery resources and supports the advancement of sustainable food systems.

## 2. Materials and Methods

### 2.1. Raw Materials, Chemicals and Reagents

Catfish (*Clarias macrocephalus* × *Clarias gariepinus*, 500–800 g/fish) were purchased in a dead and fresh condition from a local fish producer in Surat Thani Province, Thailand. After that, the fish were packed in the ice box and transported to the food processing laboratory within two hours to minimize post-harvest deterioration. Upon arrival, the fish were washed thoroughly with tap water. Evisceration and trimming were performed manually, during which the head, scales, fins, tail, and viscera were removed, while the skin was retained as an edible component, consistent with whole-body utilization. The dressed fish were rinsed again with tap water, cut into uniform pieces, and immersed in a saline solution (4% NaCl, *w*/*v*) for 30 min to reduce surface microbial contamination and improve subsequent processing quality [22]. After soaking, the fish pieces were drained and immediately transferred for cooking and drying as shown in Section 2.2.

All chemicals and reagents used in this study were of analytical grade unless otherwise stated. Sodium chloride (NaCl), hydrochloric acid (HCl), sodium hydroxide (NaOH), lithium hydroxide (LiOH), ascorbic acid, sodium citrate, performic acid, potassium bromide (KBr), sodium phosphate monobasic (NaH_2_PO_4_), sodium phosphate dibasic (Na_2_HPO_4_), sodium dodecyl sulfate (SDS), trichloroacetic acid (TCA), butylated hydroxytoluene (BHT), 2-thiobarbituric acid (TBA), acetic acid, potassium iodide (KI), sodium thiosulfate (Na_2_S_2_O_3_), phenolphthalein, and starch were obtained from Sigma-Aldrich (St. Louis, MO, USA). Concentrated nitric acid (HNO_3_, 65%), chloroform, methanol, diethyl ether, and petroleum ether were purchased from LOBA Chemie Pvt. Ltd. (Mumbai, India). Standard amino acid mixture for chromatographic quantification was obtained from Sigma-Aldrich (St. Louis, MO, USA). All microbiological culture media, including Plate Count Agar, dichloran glycerol (DG18) agar, lauryl sulfate tryptose (LST) broth, LST-MUG broth, EC medium, Rappaport–Vassiliadis (RV) medium, tetrathionate (TT) broth, xylose lysine deoxycholate (XLD) agar, Hektoen enteric (HE) agar, chromogenic agar, and buffered peptone water (BPW), were supplied by HiMedia Laboratories Pvt. Ltd. (Mumbai, India). Refined palm oil and refined soybean oil of food grade were purchased from a local commercial supplier (Surat Thani, Thailand). Deionized water and distilled water were used throughout all experiments unless otherwise specified.

### 2.2. Catfish Powder Production

**Figure 1:**
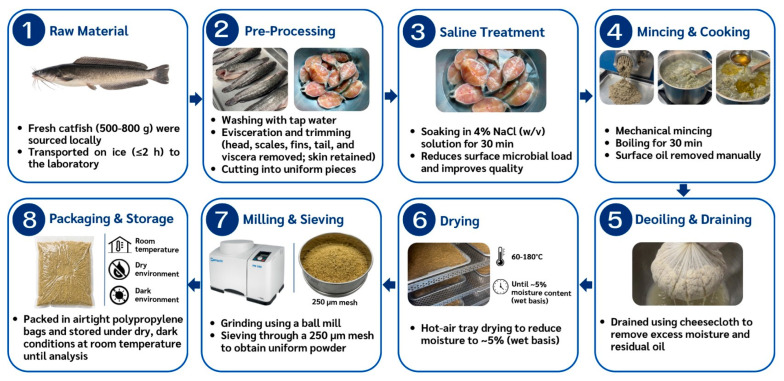
Infographic representation of CFP preparation using tray drying at different temperatures, milling and storage.

The drained catfish pieces were then minced using a lab-scale mincer (TC 12 E; ITALVER SRL, Padova, Italy) and boiled for 30 min; oil released during boiling was manually skimmed from the surface. The cooked fish was drained of excess moisture and oil using cheesecloth. The minced cooked catfish was spread uniformly on stainless steel trays at an approximate loading of 1.5 kg/m^2^ and a layer thickness of approximately 15 mm, and subjected to hot-air tray drying (Model CD-9, JST Engineering Co., Ltd., Bangkok, Thailand) at five temperatures: 60, 90, 120, 150, and 180 °C. Drying at each temperature was terminated upon reaching a final moisture content of approximately 4–6% (wet basis); the corresponding drying times were as follows: 60 °C: 16 h; 90 °C: 6 h; 120 °C: 2 h; 150 °C: 1 h; 180 °C: 50 min. This endpoint-based approach ensured that all temperature treatments were compared at equivalent final moisture contents, thereby isolating the effect of drying temperature on powder quality independently of residual water content. Following drying, the samples were cooled to ambient temperature (25 ± 2 °C) and milled using a planetary ball mill (PM 100, Retsch GmbH, Haan, Germany) at 250 rpm for 5 min per cycle with 1 min cooling intervals, using 20 stainless steel balls of 15 mm diameter. The milled material was passed through a 250 µm stainless steel mesh sieve to obtain powder of uniform particle size distribution suitable for food formulation applications. Milling and sieving were performed to standardize particle size across all drying treatments, ensuring that subsequent functional, structural, and physicochemical measurements reflected the effect of drying temperature rather than inter-treatment differences in particle size. The resulting powder was immediately transferred into airtight polypropylene bags to minimize post-milling moisture uptake. Process yield was calculated and reported for each drying temperature following the method described by Ahmad et al. [23]. The resulting CFP was transferred into airtight polypropylene bags and stored at ambient temperature (25 ± 2 °C) in a dry, dark environment pending further analysis. Figure 1 presents the infographic of the CFP preparation process. Following CFP production, the quality characteristics described below were determined.

### 2.3. Quality Characteristics

#### 2.3.1. Color and Visual Characteristics

The color characteristics of CFP were measured using a colorimeter (UltraScan VIS, HunterLab, Reston, VA, USA), where L *, a *, and b* denote lightness (0 = black, 100 = white), the red to green axis, and the yellow to blue axis, respectively. Prior to measurements, the instrument was calibrated using a white standard tile. Measurements were performed in triplicate for each powder sample placed on a flat surface. The total color difference (ΔE), chroma (C*), and hue angle (h°) were calculated in CFP using the following Equations (1)–(3):

(1) $$C^{*}=\sqrt{(a^{*}{)}^{2}+(b^{*}{)}^{2}}$$

(2) $$h{}^{\circ}=\arctan\left(\frac{b^{*}}{a^{*}}\right)$$

(3) $$\Delta E=\sqrt{\left(\Delta L^{*}{)}^{2}+(\Delta a^{*}{)}^{2}+(\Delta b^{*}{)}^{2}\right.}$$

where ΔL*, Δa*, and Δb* represent the differences in lightness, redness, and yellowness, respectively, between each sample and the CFP dried at 60 °C, which was designated as the reference treatment for ΔE calculations [24].

For macroscopic visual characterization of CFP, the samples were photographed with a digital camera (ZV-1; Sony Corporation, Tokyo, Japan), placing each sample on a uniform background to minimize environmental variability during capture. Photographs were interpreted qualitatively to describe changes in overall appearance and surface characteristics.

#### 2.3.2. Water Activity

Water activity (a_w_) of CFP was measured at ambient temperature using a dew-point water activity meter (AQUALAB 4TE, METER Group, Pullman, WA, USA), with approximately 2 g of sample used for each replication.

#### 2.3.3. pH

The pH of CFP was measured by dispersing 5 g of powder in 50 mL of distilled water, stirring until a homogeneous suspension was obtained, and reading the pH value using a calibrated digital pH meter (PB-11, Sartorius AG, Göttingen, Germany) with standard pH 4.0 and 7.0 buffer solutions (Merck KGaA, Darmstadt, Germany).

#### 2.3.4. Protein Solubility

Protein solubility at neutral pH was determined using a nitrogen solubility procedure adapted from Yin et al. [25] with some modifications. Briefly, 5 g of CFP was dispersed in 500 mL of 0.1 mol/L NaCl solution, and the pH was adjusted to 7.0 using 1.0 mol/L HCl or NaOH. The suspension was stirred for 1 h at ambient temperature and then centrifuged at 2560× *g* for 30 min. The supernatant was carefully collected, and nitrogen content in both the supernatant and the original powder was determined using a LECO nitrogen/protein analyzer (FP528, LECO corporation, St. Joseph, MI, USA). All measurements were performed in triplicate (n = 3). Protein solubility was expressed as percentage nitrogen solubility, calculated as Equation (4):

*Protein solubility* (%) = (*Nitrogen in supernatant*/*Total nitrogen in powder*) × 100 (4)

### 2.4. Proximate Composition

The proximate composition of CFP at each drying temperature was determined using a standard AOAC method [26]. Moisture content was measured gravimetrically by drying approximately 3–5 g of powder in a hot-air oven at 105 °C until a constant weight was achieved (AOAC 950.46). Crude protein content was determined using the Kjeldahl method (AOAC 928.08), with a nitrogen-to-protein conversion factor of 6.25. Crude fat was quantified by Soxhlet extraction using petroleum ether as the solvent for 8 h at 60 °C (AOAC 945.16). Ash content was determined by incineration in a muffle furnace at 550 °C until constant weight was achieved (AOAC 938.08). Total carbohydrate content was estimated according to the following Equation (5):

(5) $$\mathrm{Carbohydrate}\;(\%)=100-\left(\mathrm{Moisture}+\mathrm{Protein}+\mathrm{Fat}+\mathrm{Ash}\right)$$

All results are expressed as percentages based on dry weight basis, except for the moisture content, which was expressed as wet basis.

### 2.5. Mineral Composition

The mineral composition of CFP was determined following the method of Abbas et al. [27] with minor modifications. Briefly, approximately 1 g of each dried powder sample was mixed with 3 mL of concentrated nitric acid (HNO_3_, 65%) and heated on a hot plate until complete digestion yielded a clear solution. The digest was cooled, transferred to a volumetric flask, and made up to 100 mL with deionized water. Macro-mineral concentrations, including calcium (Ca), phosphorus (P), magnesium (Mg), sodium (Na), and potassium (K), and micro-mineral concentrations, including iron (Fe), zinc (Zn), manganese (Mn), and copper (Cu), were quantified using inductively coupled plasma optical emission spectrometry (ICP-OES; Optima 8300, Perkin Elmer, Inc., Waltham, WA, USA). All mineral analyses were performed in triplicate (n = 3) and the results are expressed as milligrams per 100 g of powder on a dry weight basis.

### 2.6. Amino Acid Profile

The amino acid profile of CFP was determined following the method of Mebratu et al. [28] with minor modifications. Approximately 2 g of each dried powder sample was hydrolyzed with 6 M hydrochloric acid (HCl) at 110 °C for 23 h. Following hydrolysis, the acid was removed by evaporation, and the dried residue was reconstituted in sodium citrate buffer (pH 2.2) and filtered through a 0.22 µm membrane filter prior to chromatographic analysis. To determine tryptophan, a separate alkaline hydrolysis was performed using 4 M lithium hydroxide (LiOH) containing 95 mM ascorbic acid as antioxidant at 110 °C for 16 h without nitrogen purging, following la Cour et al. [29]. After cooling, the hydrolysate was neutralized with 6 M HCl and filtered before analysis. To quantify cysteine and methionine, performic acid oxidation was conducted prior to acid hydrolysis to convert these sulfur-containing amino acids into stable cysteic acid and methionine sulfone derivatives, respectively. Amino acid concentrations were determined by ion-exchange chromatography with post-column derivatization using ninhydrin. Amino acid chromatography was performed using an ion-exchange analyzer (Biochrom 30+ Biochrom Ltd., Cambridge, UK). Tryptophan was analyzed separately by HPLC-MS using a HPLC-MS/MS system consisting of an Agilent 1290 infinity LC coupled with an Agilent 6490 Triple Quadrupole Mass Spectrometer (Agilent Technologies, Santa Clara, CA, USA) by following the method of la Cour et al. [29]. External calibration was performed using a certified standard amino acid mixture (Sigma-Aldrich, Cat. No. AAS18) analyzed under identical conditions. All amino acid analyses were performed in triplicate (n = 3) and the results are expressed as grams per 100 g of protein (g/100 g protein) on a dry weight basis.

### 2.7. Fatty Acid Profile

Total lipids were extracted according to the method of Bligh and Dyer [30]. Fatty acid methyl esters (FAMEs) were prepared by transesterification with BF_3_–methanol (14%, *w*/*v*) at 80 °C for 30 min. Chromatographic separation of the FAMEs was performed using a gas chromatograph equipped with a flame ionization detector (GC-FID; Agilent Technologies, Santa Clara, CA, USA) and a highly polar SP-2560 capillary column (100 m × 0.25 mm × 0.20 µm; Supelco, Bellefonte, PA, USA). Helium was used as the carrier gas at a constant flow rate of 1.2 mL/min, with a split ratio of 100:1. The injector and detector temperatures were maintained at 250 °C and 260 °C, respectively. The oven temperature was initially held at 140 °C for 5 min, ramped at 4 °C/min to 240 °C, and held at 240 °C for 15 min [31]. Individual fatty acids were identified by comparing the retention times of the sample peaks with those of a certified 37-Component FAME Mix standard (Supelco, Bellefonte, PA, USA) analyzed under identical conditions. Quantification was performed by peak-area normalization, with each fatty acid expressed as a percentage of the total integrated peak area (% of total fatty acids). The n-6/n-3 and PUFA/SFA ratios were calculated using the following Equations (6) and (7):

(6) $$n6/n3\;ratio=\frac{\sum \;n6\;PUFA}{\sum n3\;PUFA}$$

(7) $$PUFA/SFA\;ratio=\frac{\sum \;PUFA}{\sum SFA}$$

where n-6 PUFA includes linoleic acid (C18:2n-6) and arachidonic acid (C20:4n-6); n-3 PUFA includes α-linolenic acid (C18:3n-3), EPA (C20:5n-3), and DHA (C22:6n-3); PUFA represents total polyunsaturated fatty acids; and SFA represents total saturated fatty acids, all expressed as a percentage of total fatty acids.

### 2.8. Functional Properties

#### 2.8.1. Water Holding Capacity (WHC)

The WHC of CFP was determined following the centrifugation method of Nawaz et al. [21] with minor modifications. Briefly, 2 g of each powder sample was dispersed in 30 mL of distilled water in a pre-weighed centrifuge tube and allowed to stand at ambient temperature (25 ± 2 °C) for 25 min. The suspension was subsequently centrifuged at 1000× *g* for 15 min and the supernatant was carefully discarded. WHC was calculated using the following Equation (8):

(8) $$\mathrm{WHC}\;(g/g)=\;\frac{W_{r}}{W_{i}}$$

where *W_r_* represents the weight of the retained water (g) and *W_i_* represents the initial weight of the dry sample (g).

#### 2.8.2. Oil Holding Capacity (OHC)

The OHC of CFP was determined following the fat adsorption capacity method of Yin et al. [25] with minor modifications to the oil type. Briefly, 0.5 g of each powder sample was placed into a pre-weighed centrifuge tube and mixed with 10 mL of refined palm oil using a small spatula. The mixture was kept at ambient temperature (25 ± 2 °C) for 30 min with intermittent mixing every 10 min and subsequently centrifuged at 2560× *g* for 25 min. The free oil was carefully decanted and the OHC was calculated as follows (Equation (9)):

(9) $$\mathrm{OHC}\;(g/g)=\;\frac{W_{o}}{W_{i}}$$

where *W_r_* represents the weight of the retained oil (g) and *W_i_* represents the initial weight of the dry sample (g).

#### 2.8.3. Emulsifying Properties

Emulsifying activity index (EAI) and emulsion stability index (ESI) were determined according to the method of Ye et al. [24] with slight modifications. Protein solutions were prepared at 2 mg/mL in distilled water, and 15 mL of the protein solution was homogenized with 5 mL of refined soybean oil at a volume ratio of 3:1 (aqueous:oil) at 13,600 rpm for 2 min using a high-speed homogenizer. Immediately after homogenization (0 min) and at 10 min, a 50 µL aliquot was withdrawn from the bottom of the emulsion and diluted in 5 mL of 0.1% (*w*/*v*) sodium dodecyl sulfate (SDS) solution. The absorbance of the diluted emulsion was measured at 500 nm using a UV–Vis spectrophotometer. The EAI (m^2^/g) and ESI (%) were calculated using the following Equations (10) and (11):

(10) $$\mathrm{EAI}=\frac{2\times 2.303\times A_{0}\times DF}{C\times \varphi \times 10,000}$$

(11) $$\mathrm{ESI}\;(\%)=\frac{A_{10}}{A_{0}}\times 100$$

where $A_{0}$ and $A_{10}$ are the absorbance values at 0 and 10 min after emulsion formation, respectively; *DF* is the dilution factor (101); *C* is the protein concentration in the aqueous phase prior to emulsification (g/mL); and *φ* is the oil volume fraction of the emulsion (0.25).

#### 2.8.4. Apparent Viscosity

The apparent viscosity of CFP dispersions was measured at 5% (*w*/*v*) in distilled water using a Brookfield viscometer (Brookfield, WI, USA) equipped with spindle No. 2 (LV series) at 60 rpm and a constant temperature of 25 °C. Prior to measurement, samples were stirred for 30 min at ambient temperature (25 ± 2 °C) to ensure complete hydration. The spindle was allowed to rotate for 1 min before recording the reading. The results are expressed in centipoise (cP).

### 2.9. Flow, Packing, and Reconstitution Properties

#### 2.9.1. Bulk Density and Tapped Density

Bulk density (ρ_b_) and tapped density (ρ_t_) of CFP were determined following the method of Mohamed et al. [32]. Approximately 5 g of each powder sample was freely poured into a 25 mL graduated glass cylinder and the occupied volume was recorded to calculate bulk density. The same sample was then tapped manually 100 times within 1 min at a vertical drop distance of 10 mm until no further volume change was detected. Tapped density was calculated as the mass divided by the final tapped volume. Both bulk and tapped densities are expressed in grams per cubic centimetre (g/cm^3^). The Carr index (CI) and Hausner ratio (HR) were calculated from the bulk and tapped density values as indicators of powder flowability and compressibility, using the following Equations (12) and (13) [32].

(12) $$\mathrm{CI}\;(\%)=\frac{\rho_{t}-\rho_{b}}{\rho_{t}}\times 100$$

(13) $$\mathrm{HR}=\frac{\rho_{t}}{\rho_{b}}$$

Flowability was classified as follows: CI values of 0–10% and HR of 1.00–1.11 indicate excellent flow; 11–15% and 1.12–1.18 indicate good flow; 16–20% and 1.19–1.25 indicate fair flow; 21–25% and 1.26–1.34 indicate passable flow; and values above 25% and HR ≥ 1.35 indicate poor to very poor flow.

#### 2.9.2. Wettability

The wettability of CFP was assessed as the time required for the powder to become completely wetted upon contact with water. A volume of 100 mL of distilled water was placed in a 250 mL beaker. A glass funnel mounted on a ring stand was positioned 10 cm above the water surface. A 0.1 g portion of powder was placed around the outlet of the funnel, which was then opened simultaneously with the start of a stopwatch. The time elapsed until the powder was fully submerged and wetted was recorded in seconds. Shorter wettability times indicate faster water absorption and better reconstitution potential.

#### 2.9.3. Water-Dispersibility Index (WDI)

The WDI of CFP was determined with slight modifications. One gram of each powder sample was dispersed in 25 mL of distilled water and homogenized at 10,000 rpm for 5 min using a mechanical homogenizer. The dispersion was centrifuged at 1000× *g* for 5 min, and a 20 mL aliquot of the resulting supernatant was transferred into a pre-weighed Petri dish. The supernatant was dried at 105 °C for 5 h, and the WDI was expressed as a percentage using the following Equation (14):

(14) $$\mathrm{WDI}\;(\%)=\frac{W_{s}}{W_{t}}\times 100$$

where $W_{s}$ is the dry weight of dissolved solids recovered from the supernatant and $W_{t}$ is the total dry weight of the powder sample taken for analysis.

### 2.10. Structural Characterization

#### 2.10.1. Fourier Transform Infrared Spectroscopy (FTIR)

FTIR spectra of CFP samples were measured using a Spectrum Two spectrometer (PerkinElmer, Bucks, UK). Each powder sample was mixed with dry potassium bromide (KBr) at a ratio of 1:100 (*w*/*w*) and compressed into a transparent pellet using a hydraulic press. Infrared spectra were recorded over a wavenumber range of 400–4000 cm^−1^ at a spectral resolution of 4 cm^−1^ with 64 accumulated scans at ambient temperature (25 ± 2 °C). The resulting spectrum was expressed as transmittance (%).

#### 2.10.2. X-Ray Diffraction (XRD)

The crystalline structure of CFP at each drying temperature was determined by X-ray powder diffraction following the method of Ye et al. [24]. The samples were placed and flattened onto a flat glass slide and scanned over a 2θ range of 5° to 90° at a scanning rate of 2°/min using Cu Kα radiation using an XRD diffractometer (Bruker D8 Advance, Bruker AXS GmbH, Karlsruhe, Germany) at ambient temperature (25 ± 2 °C). Changes in the diffraction pattern and peak position at different drying temperatures were used to assess heat-induced structural transitions in both the protein and mineral matrices of CFP.

#### 2.10.3. Thermogravimetric Analysis

The thermal stability and decomposition behavior of CFP were evaluated using a thermogravimetric analyzer (TGA/SDTA851e; Mettler-Toledo Corporation, Zurich, Switzerland). Approximately 3–5 mg of each sample was placed in an aluminum crucible and heated from 25 to 1000 °C at a constant heating rate of 20 °C/min under a nitrogen atmosphere. Mass loss as a function of temperature was continuously recorded, and the derivative thermogravimetric (DTG) curve was generated to identify the temperatures at which the maximum decomposition rate occurred. Distinct thermal degradation stages corresponding to moisture evaporation, protein denaturation, and initial carbonization were identified and compared across treatments.

#### 2.10.4. Scanning Electron Microscopy (SEM)

The surface microstructural characteristics of the CFP samples were examined using a SEM (SU3900, Hitachi High-Tech Corporation, Tokyo, Japan). Prior to observation, the powder samples were mounted on aluminum stubs using double-sided conductive carbon tape and sputter-coated with a thin layer of gold under vacuum to ensure electrical conductivity. The SEM Images of samples were performed at an accelerating voltage of 15 kV and magnifications of 100×, and 300×.

#### 2.10.5. Particle Size, Polydispersity Index (PDI), and Zeta Potential

The effect of tray drying on the colloidal properties of CFP was assessed by measuring particle size (Z-average hydrodynamic diameter), polydispersity index (PDI), and zeta potential (ζ-potential) using dynamic light scattering (DLS) following the methods of Yin et al. [33] and Tungadi et al. [34] with modifications to sample matrix and dispersion medium. CFP samples were re-dispersed in 50 mM phosphate buffer (pH 7.0, prepared using sodium phosphate monobasic and dibasic) at a concentration of 1 mg/mL (*w*/*v*). Samples were vortexed for 1 min at maximum speed, followed by probe sonication (40 kHz, 60 W) for 5 min to ensure uniform dispersion and complete particle separation. Prior to analysis, samples were equilibrated at 25 °C ± 0.1 °C for 2 min to allow thermal stabilization. Particle size distribution analysis was performed using a Zetasizer Nano ZS instrument (Malvern Panalytical, Worcestershire, UK) equipped with a 633 nm He–Ne laser (4 mW output power) at 25 °C using non-invasive backscatter detection at a 173° scattering angle [33]. The Z-average diameter and PDI were simultaneously obtained from the autocorrelation function using cumulant analysis [34]. PDI values range from 0 (perfectly monodisperse) to 1 (highly polydisperse), with values > 0.3 indicating a broader size distribution. Zeta potential was measured using phase analysis light scattering (PALS) at a forward detection angle of 17° using the Smoluchowski model. The refractive index of the dispersant (phosphate buffer) was set to 1.330 and that of the particles to 1.590. Each sample was analyzed in triplicate, with each replicate comprising a minimum of 10 automatic runs. Zeta potential values were interpreted according to Yin et al. [33] as follows: absolute ζ-potential values ≥ ±30 mV indicate good electrostatic stabilization; values between ±10 and ±30 mV indicate moderate stability; and values < ±10 mV indicate poor stability with high propensity for aggregation and sedimentation.

### 2.11. Lipid Degradation and Oxidative Stability

#### 2.11.1. Lipid Extraction

Total lipids were extracted from each CFP sample prior to oxidative analyses using the Bligh and Dyer [30] method. Briefly, approximately 50 g of powder was homogenized in a mixture of chloroform, methanol, and distilled water at a volumetric ratio of 1:2:0.8 (*v*/*v*/*v*), and the mixture was allowed to stand for 30 min at ambient temperature (25 ± 2 °C). Chloroform and distilled water were subsequently added to achieve a final solvent ratio of 1:1:0.9 (chloroform:methanol:water, *v*/*v*/*v*), and the mixture was centrifuged at 3000× *g* for 10 min to facilitate phase separation. The lower organic phase was carefully collected and evaporated to dryness under a gentle nitrogen stream. The resulting crude lipid fraction was stored at −20 °C until further analyses.

#### 2.11.2. Free Fatty Acid Content (FFA)

FFA content was determined by titration according to the AOAC Official Method (940.28) [26] with slight modification. A 1 g of extracted lipid was dissolved in 25 mL of a neutralized ethanol–diethyl ether mixture (1:1, *v*/*v*), and two to three drops of phenolphthalein indicator solution were added. The solution was titrated against a standardized 0.1 M potassium hydroxide (KOH) solution until the first persistent pink color was observed. FFA content was expressed as a percentage of oleic acid equivalents using the following Equation (15):

(15) $$\mathrm{FFA}\;(\%\;\mathrm{oleic}\;\mathrm{acid})=\frac{V\times M\times 28.2}{W}$$

where *V* is the volume of the KOH solution consumed (mL), *M* is the molarity of the KOH solution, 28.2 is the molecular weight factor for oleic acid, and *W* is the mass of the lipid sample (g).

#### 2.11.3. Peroxide Value (PV)

PV was determined using the iodometric titration method as described by AOAC Official Method 965.33 [26]. 0.5 g of extracted lipid sample was accurately weighed into a 250 mL Erlenmeyer flask. The sample was dissolved in 30 mL of acetic acid–chloroform solution (3:2, *v*/*v*) and swirled until completely dissolved. Subsequently, 0.5 mL of saturated KI solution was added, and the mixture was allowed to stand with occasional shaking for exactly 1 min in the dark at ambient temperature (25 ± 2 °C). Following the reaction, 30 mL of distilled water was added and the mixture was titrated slowly with 0.1 M Na_2_S_2_O_3_ standard solution with vigorous shaking until the yellow iodine color had almost disappeared. A 0.5 mL of 1% starch solution was then added as an indicator, and the titration continued with vigorous shaking to release all iodine from the chloroform layer, until the blue color completely disappeared. A blank determination was simultaneously carried out under identical conditions without the lipid sample. Peroxide value was calculated using the following Equation (16):

(16) $${\mathrm{PV}\;(\mathrm{meq}\;O}_{2}/\mathrm{kg})=\frac{\left(V_{s}-V_{b}\right)\times M\times 1000}{W}$$

where $V_{s}$ is the volume of sodium thiosulfate used for the sample (mL), $V_{b}$ is the volume used for the blank (mL), *M* is the molarity of the sodium thiosulfate solution, and *W* is the mass of lipid sample (g).

#### 2.11.4. Thiobarbituric Acid Reactive Substances (TBARS)

Lipid oxidation in the CFP samples was evaluated by measuring the TBARS according to Mendes et al. [35], with slight modification. Approximately 0.5 g of powder sample was homogenized with 2.5 mL of 20% (*w*/*v*) trichloroacetic acid (TCA) containing 0.1% (*w*/*v*) butylated hydroxytoluene (BHT) to prevent further oxidation during analysis. The homogenate was centrifuged at 3000× g for 10 min. An aliquot of 2.5 mL of the resulting supernatant was mixed with an equal volume of 0.02 M thiobarbituric acid (TBA) solution prepared in 0.3% (*w*/*v*) NaOH. The mixture was heated in a boiling water bath at 100 °C for 40 min. After cooling to ambient temperature (25 ± 2 °C), absorbance was measured at 532 nm using a spectrophotometer. TBARS values were expressed as mg malondialdehyde (MDA) per kg of sample, calculated using the molar extinction coefficient of the TBA–MDA complex (155 mM^−1^ cm^−1^), Equation (17).

(17) $$TBARS\;\left(mg\frac{MDA}{kg}\right)=\frac{A_{532}\times Vtotal\times 72.06}{\varepsilon \times L\times W\times 1000}$$

where $A_{532}$ is the absorbance at 532 nm, $V_{total}$ is the total volume of the reaction mixture (mL), 72.06 is the molecular weight of MDA (g/mol), 155 is the molar extinction coefficient of the TBA–MDA complex (mM^−1^ cm^−1^), L is the path length (1 cm), and W is the sample weight (g).

### 2.12. Flavor Profile

Flavor profiles of the catfish powder samples were determined by headspace solid-phase microextraction coupled with gas chromatography/mass spectrometry (HS-SPME-GC/MS) equipped with an automatic injector, following a previously reported method with slight modifications [36]. CFP samples (3 g) were accurately weighed and transferred into 10 mL amber vials, which were subsequently sealed using aluminum caps fitted with a silicone/PTFE septum. The sealed vials were subjected to a thermal equilibration step at 40 °C for 30 min prior to volatile extraction. A divinylbenzene/carboxen/polydimethylsiloxane (DVB/CAR/PDMS) SPME fibre (50/30 µm; Supelco, Bellefonte, PA, USA) was then inserted through the septum, and the headspace volatile compounds were adsorbed onto the fiber at 40 °C for 10 min. Following adsorption, the fibre was thermally desorbed in the GC injection port at 240 °C for 7 min under a 1:10 split mode. Chromatographic separation of the extracted volatile compounds was carried out using an Rtx-Wax fused-silica capillary column (30 m × 0.25 mm; 1.0 mm i.d.). The oven temperature program was initiated at 40 °C, ramped to 200 °C at a rate of 3 °C/min with a 3 min hold, and subsequently elevated to 240 °C at 10 °C/min, where it was maintained for a further 5 min. The temperatures of the injection port, transfer line, and ion source were maintained at 240, 240, and 200 °C, respectively. Helium was used as the carrier gas at a constant flow rate of 1.5 mL/min. The mass spectrometer was operated in electron ionization mode at 70 eV, and the mass acquisition range was set at m/z 30 to 250. The volatile flavor compounds present in the CFP samples were tentatively identified by comparison of their mass spectra against entries in the NIST mass spectral library, and the results were expressed as relative peak area percentages (%).

### 2.13. Microbiological Analysis

The microbiological quality of CFP was evaluated using internationally recog-nized ISO standard procedures as described by Lekjing et al. [22]. Sample preparation involved aseptically transferring 25 g of CFP into a sterile stomacher bag, which was then diluted with 225 mL of sterile buffered peptone water (BPW) for food homogenate preparation. A stomacher (Seward, West Sussex, UK) was used to homogenize the mixture, yielding an initial 10^−1^ dilution, from which further decimal dilutions were prepared for subsequent enumeration and most probable number (MPN) analyses. Any results falling below the lower limit of detection (LOD) were designated as not detected (ND). Aerobic mesophilic bacteria were quantified for TPC determination. Each dilution was pour-plated in duplicate onto Plate Count Agar and held at 30 ± 1 °C for 72 ± 3 h. Only plates yielding between 30 and 300 colonies were considered valid for counting, and bacterial counts were reported as log_10_ CFU/g. Yeast and mold populations were assessed by spread-plating appropriate dilutions in duplicate onto dichloran glycerol (DG18) agar, a medium suitable for low-water-activity products (aw ≤ 0.95), and incubated at 25 ± 1 °C for 5–7 days, with counts expressed as log_10_ CFU/g. Coliform bacteria were quantified via the MPN approach. Serial dilutions were transferred into lauryl sulfate tryptose (LST) broth tubes and held at 37 ± 1 °C for 24 ± 3 h; tubes producing gas were subsequently inoculated into EC medium and incubated at 44 ± 0.5 °C for 48 ± 3 h to confirm the presence of coliforms, with outcomes expressed as MPN/g. The presence or absence of *Salmonella* spp. was assessed qualitatively. Pre-enrichment was carried out by incubating a 25 g test portion in 225 mL of BPW at 37 ± 1 °C for 18 ± 2 h. The pre-enriched culture was then subjected to selective enrichment in Rappaport–Vassiliadis (RV) medium at 41.5 ± 1 °C and tetrathionate (TT) broth at 37 ± 1 °C, both for 24 ± 3 h. Selective plating was performed on xylose lysine deoxycholate (XLD) agar and Hektoen enteric (HE) agar, with plates incubated at 37 ± 1 °C for 24 h. Colonies displaying characteristic *Salmonella* morphology were subjected to confirmatory biochemical testing (API 20E) and serological identification using polyvalent O and H antisera, with findings expressed as detected or not detected per 25 g. For *E. coli* detection, a presence/absence determination was applied, whereby the MPN approach was used at the level of 25 g to confirm the presence or absence of presumptive *E. coli*, with results expressed as detected or not detected per 25 g of sample. Decimal dilutions were dispensed into LST-MUG broth and incubated at 37 ± 1 °C for 24 ± 2 h, after which tubes exhibiting gas formation were regarded as presumptive coliform positives, while tubes additionally exhibiting UV fluorescence at 365 nm, indicative of β-glucuronidase activity through MUG hydrolysis, were regarded as presumptive *E. coli* positives. These were further cultured in EC broth (*Escherichia coli* broth; HiMedia Laboratories Pvt. Ltd., Mumbai, India) at 44 ± 0.5 °C for 24 ± 2 h, and final identification was carried out on chromogenic agar, with results expressed as detected or not detected per 25 g of sample.

### 2.14. Statistical Analysis

All experiments were conducted in triplicate, and the results are expressed as the mean ± standard deviation (SD). Data were subjected to one-way analysis of variance (ANOVA) to evaluate the effect of drying temperature on each measured quality parameter. When significant differences were detected (*p* < 0.05), means were compared using Duncan’s new multiple range test (DMRT) as a post hoc procedure. Differences were considered statistically significant at a confidence level of 95% (*p* < 0.05). All statistical analyses were performed using SPSS (v. 31.0) software (IBM Corp., Armonk, NY, USA).

## 3. Results and Discussion

### 3.1. Physicochemical Properties

#### 3.1.1. Color Characteristics and Appearance

The color characteristics of CFP dried by TD at different temperatures are presented in Table 1. Lightness (L*) increased significantly with increasing drying temperature, from 49.53 ± 0.41 at 60 °C to 57.64 ± 0.27 at 180 °C, indicating that the catfish powder became progressively lighter rather than darker at higher temperatures. Similar temperature-related increases in L* have been reported in dried fish and fish-based powders, where moisture removal, surface roughening, and structural rearrangement are believed to enhance light scattering and apparent brightness [37,38]. These findings suggest that, in addition to thermal reactions, physical changes in the sample structure contributed to the increased lightness observed at higher drying temperatures. Redness (a*) increased gradually from 1.94 ± 0.05 at 60 °C to 2.97 ± 0.01 at 180 °C, indicating a progressive increase in redness of the powder, consistent with heat-induced color formation through Maillard-type browning and lipid–protein interactions. In protein- and lipid-rich matrices, increases in a* are commonly associated with heat-induced color formation, including Maillard-type browning and lipid–protein interactions [39]. In the present study, the modest but significant increase in a* indicates a slight shift toward greater redness; however, given the concurrent substantial increase in L* and b*, the net colorimetric effect across the temperature range was progressively lighter and more yellow-orange powder rather than a darkened or reddish-brown product. Likewise, yellowness (b*) increased from 13.95 ± 0.37 at 60 °C to 18.14 ± 0.21 at 180 °C, indicating a stronger yellow component at elevated temperatures. This increase in b* may be related to the formation of brown pigments, transformation of natural pigments, and altered optical properties resulting from moisture loss and matrix reorganization during drying [39]. The marked rise in b* values at 180 °C, is in conjunction with the concurrent increase in L*, indicates that the powder produced at higher drying temperatures was characterized by a lighter, more intensely yellow appearance with a slight shift toward orange, as further reflected by the decrease in hue angle h°. Chroma (C*) also increased from 14.08 ± 0.38 at 60 °C to 18.38 ± 0.20 at 180 °C, reflecting a more saturated and vivid color at higher drying temperatures. This trend is consistent with the concurrent increases in a* and b*, and it indicates that the color intensity of the CFP became more pronounced as drying temperature increased, as similarly reported in thermally processed food powders. In contrast, hue angle (h°) decreased from 82.07 ± 0.11 at 60 °C to 78.32 ± 0.02 at 180 °C, signifying a slight rotation from a more purely yellow region toward a yellow-orange hue. Reductions in h° with temperature have been previously attributed to browning reactions and pigment degradation in dried marine and plant materials. Although the decrease in h° was relatively small, it supports the interpretation that increasing temperature altered the color balance of the powder toward a yellow-orange hue, consistent with the concurrent increases in L* and b*.

**Figure 2:**
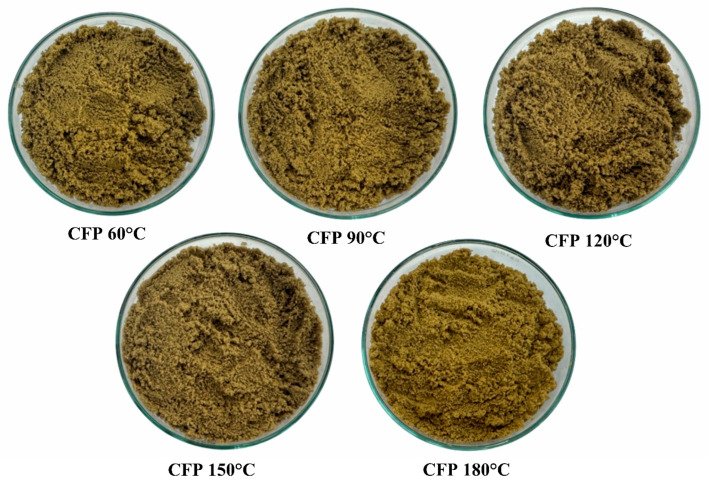
Visual appearance of CFP dried at different tray-drying temperatures.

The photographic observations were generally consistent with the instrumental measurements (Figure 2). All powders appeared yellowish-brown, but the samples dried at 150–180 °C seemed visually brighter and more chromatically intense, with a more pronounced red-yellow tone than those dried at lower temperatures. This visual pattern agrees with the higher L*, a*, b*, and C* values, along with the lower h° observed in the color analysis. Overall, increasing drying temperature produced CFP that was lighter, more red-yellow, and more chromatically intense, while slightly reducing hue angle. Therefore, the color changes observed in this cooked whole fish powder system cannot be explained solely as thermal darkening. Rather, they appear to result from the combined effects of non-enzymatic browning, pigment modification, moisture reduction, and structural changes in the surface and particles, all of which influence light scattering and perceived color [37,39]. Reactive carbonyl compounds such as malondialdehyde and 4-hydroxynonenal, generated during lipid peroxidation at elevated drying temperatures, may additionally have contributed to color development through reaction with protein amino groups to form Schiff base pigments.

#### 3.1.2. Process Yield, Water Activity, pH and Protein Solubility

Table 2 shows the changes in process yield, moisture, water activity, pH and protein solubility of CFP affected by different tray-drying temperatures. The process yield of CFP decreased progressively with increasing drying temperature, ranging from 11.66% at 60 °C to 11.41% at 90 °C, 11.11% at 120 °C, 10.91% at 150 °C, and 10.19% at 180 °C. The declining yield at higher temperatures could possibly be attributed to greater fat loss during more intensive thermal processing, as lipid exudation increases with temperature, combined with increased volatile losses at higher drying severities. These yield values have practical implications for process economics, as higher drying temperatures, while offering shorter processing times and better microbial safety, resulting in a lower mass recovery per unit of raw material. The drying times required to reach the target moisture endpoint of approximately 4–6% (wet basis) decreased markedly with increasing temperature, from 16 h at 60 °C to 50 min at 180 °C, reflecting the well-established relationship between air temperature and mass transfer rate in fish-based drying systems [7,40]. Water activity in the CFP samples was 0.42 ± 0.02 at 60 °C and remained statistically unchanged at 90 °C (0.42 ± 0.01), and then decreased significantly to 0.36 ± 0.01, 0.29 ± 0.02, and 0.23 ± 0.02 at 120, 150, and 180 °C, respectively. The pH of CFP decreased slightly but significantly from 7.88 ± 0.01 at 60 °C to 7.87 ± 0.02, 7.82 ± 0.01, 7.79 ± 0.01, and 7.73 ± 0.01 at 90, 120, 150, and 180 °C, respectively. Similar small pH reductions after drying have been reported for freshwater fish powders, where pH typically remains within a narrow range and is considered an indicator of good quality [41]. Such modest decreases are generally attributed to thermal and oxidative reactions during processing, rather than to major compositional changes, and therefore can be regarded as a minor processing-related effect [7]. The alkaline pH range observed across all treatments (7.73–7.88) is attributable to the whole-fish matrix composition of CFP, particularly the inclusion of bone and skeletal tissue contributing alkaline calcium phosphate mineral phases, alongside the release of basic non-protein nitrogen compounds during pre-cooking and thermal drying, and does not indicate spoilage, as confirmed by the microbiological data in Table 12.

Protein solubility declined from 4.12 ± 0.09% at 60 °C to 3.96 ± 0.08%, 3.71 ± 0.07%, 3.42 ± 0.06%, and 3.18 ± 0.05% at 90, 120, 150, and 180 °C, respectively. This inverse relationship with temperature is consistent with observations in fish powders and other dried protein systems, where higher thermal severity promotes denaturation, aggregation, and stronger protein–protein interactions, thereby reducing the extractable soluble fraction and functional properties [42,43]. Because the catfish had been cooked prior to drying, substantial denaturation likely occurred before the tray drying process; however, further exposure to elevated temperatures would still enhance hydrophobic and disulfide bonding and structural rearrangements, further lowering solubility [42]. Overall, increasing the drying temperature improved dehydration and stability-related attributes, but gradually diminished protein solubility and, consequently, certain functional qualities of the CFP [42]. The low absolute solubility values observed across all treatments indicate that the majority of the protein fraction exists as insoluble aggregates. This state limits the utility of CFP in applications requiring dissolved protein to exert interfacial or colloidal activity, such as beverage fortification or protein-enriched liquid foods; this constraint is directly reflected in the low EAI and declining ES values reported in Section 3.3.1. On the other hand, for the intended applications of CFP in solid or semi-solid matrices, including instant noodles, fortified flour blends, and extruded snacks, low aqueous solubility is a lesser concern, as the nutritional and structural contributions of the powder in those systems do not depend on protein dissolution. Furthermore, in vitro solubility should not be equated with in vivo digestibility; the nutritional value of CFP in terms of essential amino acid delivery remains largely intact despite the low solubility, as demonstrated by the broadly preserved amino acid profile results across treatments. The low solubility should therefore be interpreted as a processing-induced functional limitation specific to aqueous applications rather than as an indicator of reduced nutritional quality.

### 3.2. Nutritional Composition

#### 3.2.1. Proximate Composition

The proximate composition of catfish powder changed systematically with increasing tray-drying temperature from 60 to 180 °C (Table 3). Moisture content declined from 6.24 ± 0.10% to 5.91 ± 0.01%, 5.04 ± 0.04%, 5.03 ± 0.05%, and 4.75 ± 0.03% over the same temperature range. The progressive reductions in moisture content indicate that higher drying temperatures enhanced dehydration efficiency, consistent with reports that increasing air temperature accelerates heat and mass transfer and shortens drying time in fish and fish-powder systems [7,40]. From a stability perspective, the low final moisture levels and the lower a_w_ values, particularly at 120–180 °C, are favorable for shelf stability because they reduce the risk of microbial growth and quality deterioration in dried fish powders [7,11]. Fat content decreased from 22.49% at 60 °C to 22.36%, 21.93%, 20.77%, and 17.17% at 90, 120, 150, and 180 °C, respectively, representing a relative reduction of 23.7% across the full temperature range. This reduction in Soxhlet-extractable fat with increasing thermal treatment could possibly be attributed to three concurrent mechanisms rather than a single pathway. Physical drainage of melted intramuscular lipids during the pre-drying boiling step and the early phase of tray drying is considered the primary contributor to actual mass loss from the product, given that catfish intramuscular fat consists predominantly of unsaturated triglyceride species with melting points well below the processing temperatures applied in the present study; this fraction migrated physically into the cooking medium and drip drainage and is no longer present in the powder [7,44]. Oxidative fragmentation of polyunsaturated and monounsaturated fatty acids into short-chain volatile secondary compounds, including aldehydes, hydrocarbons, and ketones, may have resulted in a further fraction of lipid carbon leaving the matrix as vapor during thermal processing; this is consistent with the GC-MS volatile profile data of this study, which showed increased proportions of oxidation-derived aldehyde and hydrocarbon fractions at 150 and 180 °C, though this pathway accounts for a quantitatively smaller fraction of the total mass reduction relative to physical drainage [7,44]. Furthermore, a portion of the apparent fat reduction may not represent actual mass loss but rather analytical underestimation arising from heat-induced covalent interactions between lipid oxidation products and reactive protein groups, which can render part of the lipid fraction non-extractable by petroleum ether Soxhlet analysis; such lipid–protein adducts remain physically present in the powder but are invisible to the analytical method and would require acid hydrolysis prior to Soxhlet extraction for complete recovery [23,45]. In this context, the decrease in measured fat content does not necessarily imply complete lipid destruction but rather reflects a combination of actual mass loss through physical drainage and volatile oxidative products and a reduction in the analytically recoverable fraction under standard Soxhlet conditions. The relative contribution of each pathway cannot be definitively assigned without a mass-balance experiment that separately quantifies fat drainage, volatile lipid loss, and protein-bound lipid fractions, and this constitutes a recognized limitation of the present study.

In contrast, protein content increased from 57.63% at 60 °C to 57.90%, 59.23%, 60.58%, and 62.24% at 90, 120, 150, and 180 °C, respectively, while ash content rose from 5.28% to 8.47%, 11.01%, 11.74%, and 15.14% across the same temperature range (Table 3). Similar increases in protein and ash percentages after drying have been widely reported for dried fish and fish powders and are generally attributed to concentration effects following moisture removal rather than true formation of new protein or mineral matter [11,46]. As water is progressively removed, the relative proportion of solid constituents increases, causing protein and ash to appear more abundant on an as-is basis even if their absolute amounts remain unchanged or may even decline slightly because of thermal losses. This effect is especially relevant for powders prepared from whole fish, since the inclusion of bones and skin contributes a substantial mineral fraction to the final product. Accordingly, the rise in ash content likely reflects not only dehydration-induced concentration, but also the naturally high mineral contribution of the skeletal component in whole catfish powder [47]. The disproportionate magnitude of the ash increase relative to the modest change in residual moisture confirms that this trend was driven primarily by progressive fat loss at higher drying temperatures, which reduced the total organic fraction and amplified the proportional contribution of the thermally stable bone-derived mineral phase. However, at the highest drying temperatures, these apparent increases in protein percentage may overestimate the actual nutritional quality. While the Kjeldahl method quantifies total nitrogen, it cannot account for the loss of structural integrity caused by thermal exposure. As discussed in Section 3.2.3, sustained heating at 180 °C promotes covalent protein modifications, such as carbonylation, lysinoalanine crosslink formation, and isopeptide condensation, all of which reduce digestibility and biological value without affecting total nitrogen. The progressive decline in methionine (2.71 to 2.21 g/100 g protein) and cysteine (1.28 to 1.03 g/100 g protein) across the temperature range provides direct evidence of this thermally induced quality deterioration, despite the rising dry-basis protein values. Carbohydrate content, estimated by difference, declined from 8.36% at 60 °C to 5.36%, 2.79%, 1.88%, and 0.70% at 90, 120, 150, and 180 °C, respectively. Generally, fish-based materials, where true carbohydrate content is inherently low, this calculated fraction is highly sensitive to changes in the other proximate components, particularly moisture, protein, fat, and ash. Therefore, the apparent decline in carbohydrates should be interpreted primarily as a mathematical consequence of compositional redistribution and dehydration rather than as direct evidence of carbohydrate degradation during drying and therefore, it should be viewed as residual estimates rather than as a direct measure of carbohydrate loss [11,48].

Overall, increasing TD temperature produced CFP with lower extractable fat and a more concentrated protein- and mineral-rich composition. These patterns agree with prior work showing that drying, especially at higher temperatures, tends to concentrate protein and ash while modifying the lipid fraction in fish powders and dried fish products [44,49]. Although the observed changes are expressed as percentage increases or decreases, they primarily reflect shifts in relative proximate distribution caused by dehydration and thermal processing rather than the absolute creation of nutrients. Thus, the proximate composition results indicate that drying temperature influenced not only moisture removal, but also the apparent nutritional profile and compositional balance of the final powder. From a product development perspective, the progressive increase in protein percentage positions higher-temperature-dried CFP as a more protein-concentrated ingredient, advantageous for fortification or supplement applications where declared protein content is a key formulation criterion. On the other hand, this apparent compositional benefit should be considered alongside the preferential loss of polyunsaturated fatty acids at higher temperatures (Section 3.2.4) and the protein quality limitations discussed in Section 3.2.3. Among the tested conditions, 120 °C represented the most favorable balance between adequate protein concentration (59.23%) and limited lipid and protein quality deterioration.

#### 3.2.2. Mineral Composition

The mineral composition of CFP was significantly affected by drying temperature (Table 4). Calcium increased from 748.25 mg/100 g at 60 °C to 793.54 mg/100 g at 180 °C, with similar gradual increases observed for phosphorus, potassium, sodium, magnesium, and manganese. In contrast, iron and zinc showed slight declines across the same temperature range. Overall, most minerals exhibited small but consistent increases with increasing drying temperature, whereas a few elements decreased modestly, indicating that thermal treatment influenced individual minerals differently rather than uniformly. These patterns are consistent with reports that drying primarily concentrates minerals through moisture removal rather than generating true gains in mineral mass. Sroy et al. [7] reported that, in small freshwater fish dried between 50–80 °C, mineral contents, including calcium, phosphorus, iron, zinc, and manganese, increased on a wet-weight basis but remained essentially unchanged when expressed on a dry basis, confirming that apparent increases mostly reflect water loss. Similar concentration effects of thermal treatment on sodium, calcium, potassium, iron, and zinc have also been observed in sundried tilapia subjected to salt–heat pre-treatments [50]. Because the CFP was produced from whole fish, including bone and skin, the mineral profile was strongly influenced by skeletal tissues. Fish bone- and backbone-derived powders are characteristically rich in calcium and phosphorus, with Ca and P forming the dominant mineral phases in bone matrices and side-stream powders. This helps explain the prominence of calcium and phosphorus in the CFP and their relatively stable or slightly increasing concentrations at higher drying temperatures. The small increases in potassium, sodium, and magnesium with temperature are also consistent with literature showing that drying and processing conditions may alter the relative distribution of minerals among muscle, bone, and viscera rather than causing major absolute mineral losses, particularly when leaching into water is minimal [51,52]. In contrast, the slight reductions in iron and zinc may be attributed to analytical variation, differences in tissue distribution, or minor changes in the measurable fraction after heating. Similar modest decreases or variable trends have been reported for some minerals during cooking and smoking of fish fillets and whole fish [52]. Overall, tray drying from 60–180 °C produced only modest changes in the mineral composition of whole catfish powder. Most macro- and microelements showed slight increases consistent with concentration and compositional redistribution effects, while iron and zinc decreased slightly; nonetheless, the overall mineral profile of the whole-fish powder remained largely preserved across the tested temperature range [7,27,50]. The calcium content across all treatments (748–794 mg/100 g) is particularly noteworthy, reflecting the substantial skeletal contribution of whole-fish processing and positioning CFP as a potential calcium fortification ingredient, especially in food systems where dairy-derived calcium is limited [47]. The modest declines in iron (18.95 to 17.61 mg/100 g) and zinc (5.84 to 5.51 mg/100 g) are nutritionally relevant given that these micronutrients are frequently limiting in dietary contexts where CFP-fortified products are most likely to be consumed; for applications prioritizing iron and zinc delivery, lower drying temperatures (60–90 °C) are therefore preferable.

#### 3.2.3. Amino Acid Profile

Amino acid profile in the CFP samples is presented in Table 5. Overall, the TD process only moderately altered the amino acid composition of CFP, indicating that the overall protein fraction remained relatively stable across the studied temperature range. This is consistent with reports that thermal processing may modify individual amino acids without causing a drastic decline in total protein quality when processing conditions are controlled [28]. Among the essential amino acids, leucine remained the most abundant, followed by lysine and arginine, whereas tryptophan was the least abundant. With increasing drying temperature, several essential amino acids, including leucine, isoleucine, valine, methionine, phenylalanine, and arginine, showed gradual reductions, with methionine decreasing from 2.71 to 2.21 g/100 g protein. Similar preferential losses of sulfur-containing amino acids at elevated temperatures have been reported in both plant and fish matrices, reflecting their particular susceptibility to thermal and oxidative degradation. Decreases in threonine, serine, tyrosine, and the branched-chain amino acids further suggest that drying intensity selectively affected heat-sensitive residues rather than causing uniform degradation across all amino acids, in agreement with the amino acid-specific temperature sensitivity reported for dried soy and fish products [28]. Lysine exhibited an atypical pattern, increasing from 6.21 g/100 g protein at 60 °C to 6.78 g/100 g protein at 150 °C, followed by a slight decline at 180 °C. Mechanistically, lysine is generally considered one of the most heat-labile amino acids because its ε-amino group readily participates in Maillard reactions, which can reduce biologically available lysine even when total lysine measured after acid hydrolysis appears unchanged [53]. Therefore, the observed increase is more plausibly explained by differences in drying time, cumulative exposure, and normalization on a protein basis than by a true improvement in lysine preservation [28,54]. At drying temperatures of 150–180 °C, protein quality changes extend beyond simple denaturation and concentration effects. Sustained thermal exposure at these temperatures promotes β-elimination of cystine disulfide bonds (generating dehydroalanine, which reacts with lysine to form lysinoalanine), formation of isopeptide crosslinks through ε-(γ-glutamyl) lysine condensation, and progressive carbonylation of aliphatic amino acid side chains (valine, leucine, isoleucine, threonine), all of which reduce protein digestibility and biological value in ways not captured by total amino acid analysis after acid hydrolysis [55,56]. The observed declines in methionine (from 2.71 to 2.21 g/100 g protein) and cysteine (from 1.28 to 1.03 g/100 g protein) across the temperature range are consistent with thermal and oxidative degradation of sulfur-containing amino acids and support the interpretation that protein nutritional quality deteriorated progressively at higher drying temperatures beyond what the increasing protein percentage alone would indicate.

Among the non-essential amino acids, glutamic acid was the most abundant, followed by aspartic acid and glycine. Progressive reductions in glutamic acid, aspartic acid, alanine, serine, tyrosine, and cysteine with increasing temperature are consistent with documented thermal degradation pathways and with the known high heat sensitivity of sulfur-containing amino acids, particularly cysteine. In contrast, glycine and proline increased with temperature, reaching 6.75 and 3.54 g/100 g protein at 180 °C, respectively. Because the powder was prepared from whole fish and included skin and bone, the protein matrix likely contained a substantial collagen fraction. Sun et al. [57] reported that collagen is a major component of fish skin. Collagen-rich fish tissues are characteristically enriched in glycine and proline, and processing may increase their apparent contribution in the final amino acid profile [58]. Regarding the observed trend in tryptophan (0.81 to 0.97 g/100 g protein), the increase is not an analytical artifact. Tryptophan was quantified using a specialized alkaline hydrolysis method (4 M LiOH with 95 mM ascorbic acid at 110 °C for 16 h) following la Cour et al. [29], which is the standard procedure for reliable tryptophan quantification in food proteins. This increase likely reflects compositional shifts in the total protein fraction rather than tryptophan synthesis; for example, the progressive reduction in extractable lipid content during high-temperature drying increases the relative mass fraction of the protein component during normalization. Given that the magnitude of the increase (0.16 g/100 g protein) is relatively small, the results indicate that tryptophan was broadly preserved across the 60–180 °C range, consistent with the stability observed in most other amino acids (Table 5). Overall, the amino acid profile of tray-dried CFP was broadly preserved across 60–180 °C, with selective reductions in heat-sensitive and sulfur-containing amino acids and an apparent enrichment of collagen-associated glycine and proline.

#### 3.2.4. Fatty Acid Profile

Tray drying at increasing temperatures progressively altered the fatty acid profile of CFP, shifting the lipid fraction toward greater saturation (Table 6). At 60 °C, MUFA represented the largest fraction of total identified fatty acids (37.67 ± 0.38%), followed by SFA (35.63 ± 0.41%) and PUFA (26.70 ± 0.41%). This distribution is consistent with the known lipid composition of *Clarias* species, in which oleic acid (C18:1n-9) and palmitic acid (C16:0) are typically dominant [59]. As drying temperature increased from 60 to 180 °C, SFA increased steadily from 35.63% to 42.37%, whereas MUFA decreased from 37.67% to 35.78% and PUFA decreased from 26.70% to 21.85%. These results indicate that higher tray-drying temperatures preferentially affected unsaturated fatty acids, while saturated fatty acids became relatively more prominent in the final powder [23]. The increase in SFA should therefore be interpreted mainly as a relative enrichment effect arising from the greater loss of unsaturated fractions rather than as a true synthesis of saturated lipids during drying. Among the saturated fatty acids, palmitic acid (C16:0) was the predominant fatty acid across all treatments and increased significantly from 25.12 ± 0.23% at 60 °C to 30.55 ± 0.36% at 180 °C. Stearic acid (C18:0) and myristic acid (C14:0) also showed gradual increases, rising from 7.48 to 8.43% and from 2.51 to 2.79%, respectively. This pattern suggests that SFA were comparatively more stable during heating, and their apparent increase likely reflects the preferential degradation or loss of more unsaturated lipids [11,46]. Because the powder was prepared from whole fish, including bone and skin, the observed fatty acid composition likely reflects both muscle lipid and lipids associated with other tissues, which may further influence the relative proportions of the major fatty acid classes. The concurrent decrease in total extractable fat from 22.49% at 60 °C to 17.17% at 180 °C supports the interpretation that thermal treatment caused net lipid loss, with unsaturated lipids being more susceptible to oxidative deterioration and analytical recovery loss than saturated lipids [16,60]. Among the monounsaturated fatty acids, oleic acid (C18:1n-9) remained the most abundant MUFA, followed by palmitoleic acid (C16:1n-7) and gondoic acid (C20:1n-9). Oleic acid decreased significantly from 28.15 ± 0.25% at 60 °C to 26.57 ± 0.36% at 180 °C, with no significant difference between 60 and 90 °C but significant reductions beginning at 120 °C. Palmitoleic acid also declined from 8.04 ± 0.10% to 7.55 ± 0.12%, while gondoic acid decreased slightly from 1.48% to 1.41%. These changes indicate that MUFAs were moderately affected by drying temperature, although their losses were less severe than those observed for PUFA. This is expected because MUFAs contain only one double bond and are generally more resistant to thermal oxidation than highly unsaturated fatty acids. Nevertheless, the steady decline in oleic acid and the smaller decreases in other MUFAs show that prolonged exposure to elevated drying temperatures still altered the MUFA fraction of the lipid profile.

The PUFA fraction was the most sensitive to increasing drying temperature. Linoleic acid (C18:2n-6, LA) was the most abundant PUFA and decreased from 15.08 ± 0.17% at 60 °C to 13.33 ± 0.20% at 180 °C. α-Linolenic acid (C18:3n-3, ALA) showed a similar decline from 1.98% to 1.57%. Among the long-chain PUFAs, arachidonic acid (C20:4n-6, AA), eicosapentaenoic acid (C20:5n-3, EPA), and docosahexaenoic acid (C22:6n-3, DHA) all decreased significantly with increasing temperature. AA declined from 3.51 ± 0.08% to 2.67 ± 0.10%, EPA from 1.52 ± 0.04% to 1.08 ± 0.05%, and DHA from 4.61 ± 0.10% to 3.20 ± 0.13%. The relative loss order of DHA > EPA > AA is consistent with the known susceptibility of fatty acids to oxidation according to degree of unsaturation, since molecules with more double bonds contain more bis-allylic sites and are therefore more vulnerable to radical attack and chain propagation [7,23]. This explains why DHA, the most unsaturated fatty acid in the profile, showed the steepest decline across the drying range, followed by EPA and then AA. The nutritional indices derived from the fatty acid composition further demonstrate the decline in lipid quality with increasing drying temperature. The total Σn-3 PUFA decreased from 8.11% at 60 °C to 5.85% at 180 °C, while Σn-6 PUFA declined from 18.59% to 15.48%. As a result, the n-3/n-6 ratio decreased from 0.44 to 0.37, indicating a progressive reduction in the relative contribution of health-promoting n-3 fatty acids [14]. The PUFA/SFA ratio also declined from 0.75 to 0.52, reflecting the combined effect of decreasing PUFA and increasing SFA. Although this ratio remained above 0.50 across all treatments, suggesting that the powder retained an acceptable overall lipid profile, the downward trend clearly shows that higher drying temperatures reduced the nutritional value of the lipid fraction. In practical terms, the results indicate that temperatures above 120 °C caused noticeable deterioration in fatty acid quality, particularly through losses in long-chain n-3 PUFA such as DHA and EPA [23]. Overall, the fatty acid data show that tray drying from 60 to 180 °C progressively shifted whole catfish powder toward a more saturated lipid profile, with SFA increasing and both MUFA and PUFA declining. Among the tested conditions, 120 °C appears to provide a reasonable balance between drying efficiency and lipid quality preservation, as it limits the excessive losses of PUFA while still producing a stable powder with relatively favorable fatty acid composition.

### 3.3. Functional Properties

#### 3.3.1. Water Holding, Oil-Binding, Emulsifying, and Viscosity Properties

The functional properties of CFP were selectively affected by drying temperature, with different parameters showing distinct responses to increasing thermal severity (Table 7). Water holding capacity (WHC) decreased significantly from 2.13 ± 0.16 g/g at 60 °C to 2.01 ± 0.11 g/g at 90 °C, and then more markedly to 1.79 ± 0.05, 1.74 ± 0.08, and 1.65 ± 0.05 g/g at 120, 150, and 180 °C, respectively. This decline indicates that higher drying temperatures reduced the ability of the powder to absorb and retain water. It is due to stronger thermal treatment that can promote protein denaturation, aggregation, and structural collapse, thereby reducing the availability of hydrophilic groups and limiting the hydration capacity of the powder matrix [61]. In addition, severe drying may reduce porosity and swelling ability, further decreasing water retention [42]. A similar trend was observed for oil holding capacity (OHC), which decreased from 1.13 ± 0.04 g/g at 60 °C to 1.12 ± 0.17, 1.04 ± 0.10, 0.99 ± 0.07, and 0.90 ± 0.05 g/g at 90, 120, 150, and 180 °C, respectively. The reduction in OHC suggests that increasing drying temperature negatively affects the oil-binding properties of the powder. This may be attributed to heat-induced conformational changes, aggregation, or compaction of proteins and other matrix components, which could reduce the accessibility of hydrophobic sites or the structural spaces required to entrap oil [62]. In a fish powder system that contains muscle proteins, collagen-associated materials, minerals, and residual lipid fractions, stronger drying conditions may intensify inter-molecular interactions and produce a denser structure with reduced oil retention capacity [63]. A comparable declining trend was observed for the emulsion activity index (EAI), which decreased significantly with increasing drying temperature. The sample dried at 60 °C exhibited the highest EAI of 52.14 ± 1.23 m^2^/g, which decreased to 48.87 ± 0.94 m^2^/g at 90 °C and further to 46.32 ± 1.45, 42.18 ± 1.67, and 38.54 ± 2.01 m^2^/g at 120, 150, and 180 °C, respectively. This reduction indicated that the capacity of the powder to generate new interfacial area during emulsification was progressively impaired at higher drying temperatures. It could possibly be attributed to heat-induced protein denaturation and aggregation, which reduced protein solubility and surface activity and thereby limited the rapid adsorption and unfolding of the matrix components at the oil–water interface [62].

In contrast, emulsion stability (ES) decreased significantly with increasing drying temperature. The highest ES was recorded at 60 °C (57.14 ± 1.32%), which declined progressively to 55.38 ± 0.87% at 90 °C, 52.69 ± 0.73% at 120 °C, 48.23 ± 0.92% at 150 °C, and 42.10 ± 1.07% at 180 °C. This declining trend parallels the decrease in EAI and protein solubility across the same temperature range and reflects the progressive loss of interfacial film quality with increasing thermal severity. In pre-cooked fish protein systems, emulsion stability depends on the ability of solubilized surface-active proteins to adsorb, spread, and rearrange at the oil–water interface to form a viscoelastic continuous film. More severe drying temperatures promote irreversible aggregation and cross-linking, reducing the fraction of protein available to support interfacial adsorption and generating compact, rigid aggregates with limited capacity for interfacial spreading and film cohesion [62,64]. Although larger particles can in principle adsorb more strongly at the interface, the dominant mechanism in this system is molecular film formation rather than particle-type stabilization; under that mechanism, structural rigidity and insolubility are liabilities rather than assets. The concurrent decline in protein solubility, EAI, and ES with increasing drying temperature collectively confirms that higher thermal severity impaired the emulsifying functionality of whole catfish powder across all measured dimensions. Viscosity also decreased significantly as drying temperature increased, falling from 7.16 ± 0.13 cP at 60 °C to 6.87 ± 0.10 cP, 6.71 ± 0.08 cP, 6.67 ± 0.09 cP, and 6.32 ± 0.09 cP at 90, 120, 150, and 180 °C, respectively. This progressive reduction suggests that powders produced at higher drying temperatures formed less viscous dispersions. Viscosity in such systems is closely related to hydration, swelling, and the ability of macromolecules and particles to form a structured network in the aqueous phase [65]. Therefore, the decrease in viscosity is consistent with the reduction in WHC and suggests that higher drying temperatures weakened the structural and hydration-related functionality of the powder [42]. The declining trends across all functional parameters share a common mechanistic basis: heat-induced protein denaturation and aggregation progressively reduced the accessible polar surface area responsible for water binding (WHC), collapsed the inter-protein structural spaces required for oil entrapment (OHC), and diminished the soluble protein fraction available for interfacial adsorption (EAI and ES), while simultaneously weakening the hydration network governing viscosity. Overall, the results showed that increasing drying temperature progressively impaired all measured functional properties of CFP, with WHC, OHC, EAI, ES, and viscosity all declining with increasing thermal severity, reflecting the cumulative impact of heat-induced protein structural changes on the functional performance of the powder.

#### 3.3.2. Flow, Packing, and Reconstitution Properties

The flow, packing, and reconstitution properties of CFP affected by different tray-drying temperatures are shown in Table 8. The Carr Index (CI) decreased from 12.00 ± 0.21% at 60 °C to 8.74 ± 0.15% at 90 °C, and then increased slightly to 9.72 ± 2.29%, 10.14 ± 0.00%, and 10.54 ± 1.27% at 120, 150, and 180 °C, respectively. A similar pattern was observed for the Hausner ratio (HR), which decreased from 1.12 ± 0.00 at 60 °C to 1.09 ± 0.00 at 90 °C, followed by values of 1.10 ± 0.02, 1.10 ± 0.00, and 1.11 ± 0.01 at 120, 150, and 180 °C, respectively. These results indicate that the 90 °C treatment produced the most favorable flow-related behavior among the tested samples. Since lower CI and HR values are generally associated with better powder flowability and lower cohesiveness, the decrease observed at 90 °C suggests improved flow characteristics relative to the 60 °C treatment, whereas the slight increase at higher temperatures indicates a partial loss of that advantage. However, because both CI and HR remained within a relatively narrow and low range across all treatments, it indicates that CFP can still be considered to have generally acceptable flow behavior. The non-monotonic CI pattern could possibly be attributed to competing effects of drying temperature on particle surface properties. At 60 °C, the prolonged drying duration and comparatively higher residual moisture promoted inter-particle cohesion through partially solvated protein surfaces, contributing to the highest CI. At 90 °C, faster moisture removal produced drier, less cohesive particles, yielding the minimum CI. At temperatures above 90 °C, progressive protein denaturation, aggregate densification, and surface hardening with increasing thermal severity gradually increased inter-particle friction, accounting for the modest upward trend in CI at 120–180 °C.

In contrast, bulk density and tapped bulk density were not significantly affected by drying temperature. Bulk density remained within the range of 0.39–0.40 g/mL, while tapped bulk density remained within 0.43–0.45 g/mL across all treatments. These results indicate that drying temperature had little influence on the loose and compact packing characteristics of the powder, suggesting that the gross physical arrangement of the particles remained relatively stable despite changes in drying severity. This is in accordance with the study of Mohamed et al. [42]. Wettability showed a clear deterioration as drying temperature increased. The wetting time increased progressively from 8.50 ± 2.21 s at 60 °C to 10.88 ± 0.63 s, 12.62 ± 2.03 s, 15.54 ± 2.99 s, and 16.82 ± 2.38 s at 90, 120, 150, and 180 °C, respectively. The increase in wetting time indicates that powders dried at higher temperatures became less readily penetrated by water [59]. Severe thermal treatment may modify powder surface characteristics, reduce surface hydrophilicity, promote stronger inter-particle aggregation, or produce a denser particle structure, all of which can delay water penetration during reconstitution [63]. The water-dispersible fraction showed the opposite trend, decreasing from 44.20 ± 1.10% at 60 °C to 41.85 ± 1.25%, 37.90 ± 1.42%, 34.75 ± 1.36%, and 31.60 ± 1.28% at 90, 120, 150, and 180 °C, respectively. This decline suggests that higher drying temperatures reduced the proportion of powder components recoverable in the aqueous phase. This could possibly be attributed to severe thermal treatments, which promote protein denaturation, aggregation, and reduced dispersibility, thereby lowering the fraction of material that readily enters or remains in the aqueous phase during reconstitution [66]. Since the fish was cooked before drying, the protein system was likely already partially denatured before dehydration, and further thermal exposure during drying may have intensified insolubilization. Overall, the results show that drying temperature had only minor effects on bulk density and tapped bulk density, moderate effects on CI and HR, and a more pronounced negative effect on wettability and the water-dispersible fraction.

### 3.4. Structural Characteristics

#### 3.4.1. FTIR

**Figure 3:**
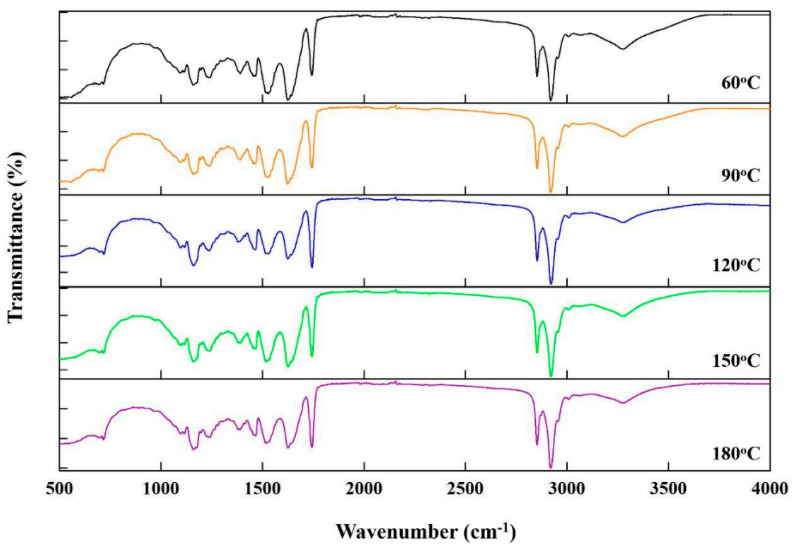
FTIR spectra of CFP dried at different tray-drying temperatures.

The FTIR spectra of CFP tray-dried at different temperatures showed a broadly similar spectrum across all treatments (Figure 3), indicating that the major chemical functional groups of the powder were retained despite differences in drying temperature. All samples exhibited a broad absorption region in the ~3200–3400 cm^−1^ range, bands near ~2920 and ~2850 cm^−1^, a prominent region around ~1700–1500 cm^−1^, and multiple bands in the fingerprint region below 1500 cm^−1^. The preservation of these main bands suggests that tray drying did not fundamentally alter the overall chemical class composition of the catfish powder, but rather affected the relative expression, molecular interactions, and structural organization of existing components [8,67]. The broad band in the ~3200–3400 cm^−1^ region is reasonably associated with overlapping O–H and N–H stretching vibrations, which may reflect bound water, proteinaceous components, and hydrogen-bonded structures within the powder matrix [8]. Although this broad region remained present in all samples, subtle changes in intensity and shape with increasing drying temperature suggest progressive dehydration and modification of hydrogen-bonding interactions. This interpretation is consistent with the reduction in moisture content and water activity observed in the physicochemical properties [68]. The bands near ~2920 and ~2850 cm^−1^ are consistent with aliphatic C–H asymmetric and symmetric stretching vibrations and are plausibly related to lipid-associated and other hydrocarbon-containing components of the whole-fish matrix [8]. These peaks remained detectable in all treatments, suggesting that the main lipid-associated structural features were preserved across the drying conditions. However, slight changes in band shape or relative expression at higher drying temperatures may indicate modest alterations in the molecular environment of lipid-containing components rather than complete chemical loss [69]. The region between ~1700 and 1500 cm^−1^ is particularly important because it includes the amide I and amide II bands, which are commonly associated with protein-rich materials. The band at 1700–1600 cm^−1^ encompasses the Amide I region (~1650 cm^−1^, primarily C=O stretching of peptide bonds), alongside contributions from other carbonyl-containing functional groups including carboxylic acids and carbonyl species from lipid oxidation products in the upper portion of this range. The amide II band in the ~1500–1600 cm^−1^ region is generally associated with N–H bending and C–N stretching vibrations [8,67]. In the present study, these bands remained present across all drying temperatures, indicating the continued presence of proteinaceous structures in the powder. The relatively similar appearance of these bands at 60 and 90 °C suggests that protein-associated molecular features were largely preserved under moderate drying conditions. At higher temperatures, particularly 150 and 180 °C, slight changes in the amide region became more apparent, suggesting moderate heat-induced conformational modification, altered hydrogen bonding, or partial denaturation of protein components rather than complete structural breakdown [70]. The fingerprint region below 1500 cm^−1^ showed multiple absorption bands in all samples, reflecting the chemically complex nature of the whole-fish powder. Because the powder was produced from cooked whole fish including muscle, skin, and bone, this region is expected to contain overlapping contributions from proteins, collagen-associated materials, lipids, and other matrix constituents [71]. Minor differences in peak sharpness and intensity suggest thermal reorganization within this biomolecular system rather than formation of new chemical groups [69]. Overall, the FTIR results indicate that tray drying at different temperatures preserved the principal functional-group profile of catfish powder while causing only moderate changes in band intensity and spectral expression.

#### 3.4.2. XRD

**Figure 4:**
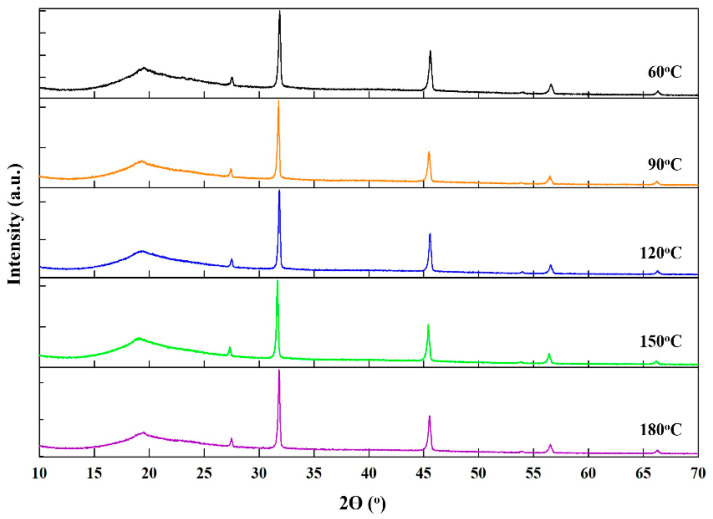
XRD of CFP dried at different tray-drying temperatures.

The XRD patterns of CFP dried at different temperatures showed a similar overall diffraction profile, indicating that the structural characteristics of the powder were generally preserved across treatments (Figure 4). All samples exhibited a broad diffraction halo in the region of approximately 2*θ* = 18–24°, together with several sharp reflections at higher angles, particularly near 2*θ* ≈ 32°, 45°, 56–57°, and 66°. The sharp reflections at 2θ ≈ 32° are assigned to the (211) and (300) planes of hydroxyapatite [Ca_10_(PO_4_)_6_(OH)_2_; JCPDS Card No. 09-0432], and the additional reflections at 2θ ≈ 45°, 56–57°, and 66° are consistent with higher-order reflections of the same phase, indicative of a poorly crystalline biological apatite derived from the skeletal fraction [72]. The coexistence of a broad halo and sharp peaks indicates that the powder contained both amorphous or weakly ordered components and crystalline phases, which is typical for materials containing an organic matrix and mineral-rich fractions. This type of mixed amorphous–crystalline pattern is scientifically reasonable for a whole-fish powder prepared from muscle, skin, and bone, where organic components and skeletal minerals are expected to coexist [73]. Although the general diffraction pattern was similar in all samples, slight differences in relative peak prominence were observed with increasing drying temperature. The broad amorphous halo appeared more pronounced in the powders dried at 60–120 °C, whereas the samples dried at 150 and 180 °C showed somewhat greater prominence of the sharp crystalline reflections relative to the background. These observations suggest that drying temperature caused modest structural changes but did not fundamentally alter the mixed amorphous–crystalline nature of the catfish powder. A shift in the balance between amorphous and crystalline features with temperature is consistent with reports where higher temperatures modified crystallinity and peak intensity in calcium phosphate materials derived from fish bone [74,75]. The reduced prominence of the broad halo and the relatively stronger appearance of crystalline peaks at higher drying temperatures may indicate a lower relative contribution of amorphous material or greater apparent prominence of crystalline components, and furthermore, elevated thermal treatment can promote structural reorganization, particle agglomeration, and changes in diffraction peak intensity in mineral-rich biological systems [74,76]. Such behavior is mechanistically plausible because higher drying temperatures can reduce residual moisture, promote matrix compaction, and modify the organization of the organic fraction, thereby affecting the relative expression of amorphous and crystalline features in the diffraction pattern [77]. The broad diffraction halo at 2θ = 18–24° is attributed primarily to thermally denatured proteins and disrupted collagen, both of which produce diffuse amorphous scattering in this angular region in fish-derived biological materials; a minor contribution from disordered lipid phases cannot be excluded. Overall, the XRD results indicate that tray drying preserved the general structural pattern of the CFP, while causing only modest temperature-dependent variations in the balance between amorphous and crystalline features.

#### 3.4.3. TGA

**Figure 5:**
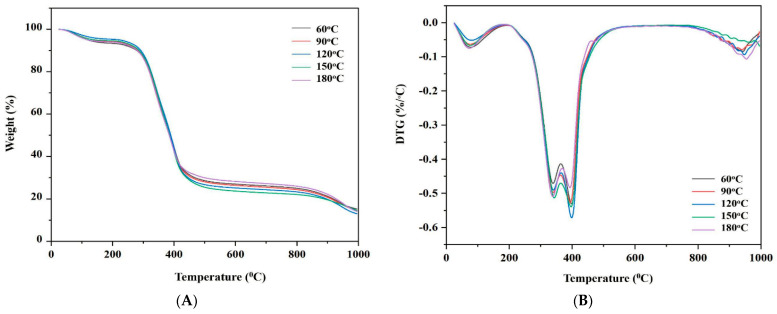
TGA (**A**) and DTG (**B**) of CFP dried at different tray-drying temperatures.

CFP exhibited a reproducible three-stage thermal decomposition profile across all different tray-drying temperatures, with the overall pattern governed by the chemical complexity of the whole-fish matrix rather than by drying temperature alone (Figure 5A). All samples began near 100% initial mass and progressed through an initial low-temperature event, a dominant intermediate decomposition stage, and a final gradual mass-loss region terminating in a substantial mineral-rich residue. The initial stage, confined to temperatures below approximately 150 °C, accounted for approximately 3–4% mass loss and is attributable to the release of residual moisture and low-molecular-weight volatile species retained within the powder matrix. This observation aligns with the general interpretation of moisture/volatile loss in protein-rich biological powders, where the magnitude of early mass loss tracks pre-drying moisture content and moisture–structural state rather than dictating subsequent degradation pathways [78]. The dominant decomposition event occurred between approximately 250 and 450 °C and accounted for approximately 66–70% of total mass loss across all samples. This stage reflects the concurrent thermal degradation of the principal organic constituents of the whole-fish matrix, including myofibrillar and sarcoplasmic proteins, lipid-associated components, and collagen-derived material. DTG profiles within this region resolved into two overlapping minima in the 320–420 °C range, indicating that decomposition proceeded through at least two thermally distinct but concurrent processes rather than a single unified reaction, a pattern characteristic of heterogeneous biological matrices with overlapping degradation of macromolecular components. Among the treatments, the 120 °C sample exhibited the most pronounced DTG minimum, suggesting a marginally higher peak decomposition rate, whereas the remaining treatments showed comparable derivative profiles (Figure 5B). These inter-treatment differences were minor and did not indicate mechanistically distinct degradation pathways but rather reflected modest variations in matrix organization resulting from differences in drying severity [78]. At temperatures above the principal decomposition zone, a third stage of gradual continued mass loss of approximately 10–13% was observed between 600 and 1000 °C, attributable to thermal decomposition of residual calcium carbonate and partial dehydroxylation of hydroxyapatite, both of which are characteristic of bone-derived mineral phases at elevated temperatures. All samples retained a final residue of approximately 15–18% at 1000 °C, reflecting the thermally stable inorganic fraction of the powder, dominated by bone-derived mineral phases such as hydroxyapatite and other calcium phosphate compounds that persist well beyond the organic decomposition range. This interpretation is directly supported by the high ash and mineral content measured in the proximate and mineral composition analyses, where whole-bone inclusion was identified as the primary contributor to the mineral fraction of the powder. Overall, the TGA and DTG data collectively demonstrate that tray drying across the 60–180 °C range did not alter the fundamental thermal decomposition mechanism of whole catfish powder. The treatment-related differences observed in mass retention and decomposition rate were modest and superimposed on a conserved degradation framework dictated by the intrinsic chemical composition of the whole-fish matrix.

#### 3.4.4. SEM

**Figure 6:**
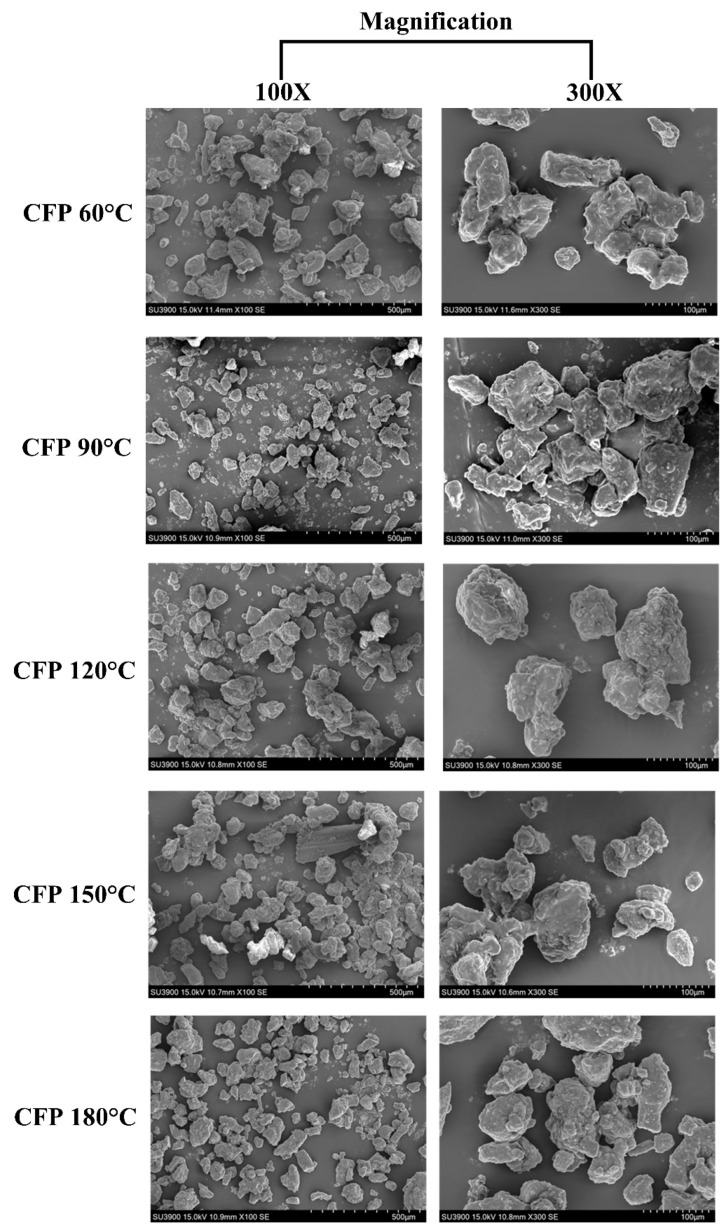
Microstructural changes of CFP dried at different tray-drying temperatures.

SEM micrographs of CFP at 100× and 300× magnifications showed that drying temperature influenced surface morphology and particle organization (Figure 6). Across all treatments, the powders consisted of irregular, non-spherical, and heterogeneous particles with fractured, flake-like, and agglomerated structures. Elavarasan and Shamasundar [79] reported that oven-dried fish protein hydrolysate powders showed irregularly broken and flake-like particle morphology; however, the study of Gómez-Guillén et al. [15] found that heat-dried marine protein matrices produced angular, fragmented, and structurally heterogeneous particles rather than smooth or spherical ones, both of which are consistent with the morphology observed in the present study. At 60 °C, the powder showed relatively coarse morphology, with larger flake-like fragments and broad irregular particles at 100×; at 300×, surfaces appeared moderately rough with layered fractured edges, and the matrix appeared less compact than in samples dried at higher temperatures. At 90 °C, the powder displayed a more heterogeneous particle distribution, with abundant smaller broken fragments at 100× and rough-surfaced irregular particles at 300×, suggesting that this drying temperature rendered the matrix more brittle and susceptible to fracture during milling. At 120 °C, particles appeared more condensed, with clustered aggregates at 100× and compact aggregates with uneven surfaces at 300×. Elavarasan and Shamasundar [79] reported similar intensified matrix shrinkage and densification in fish-derived powders subjected to more severe thermal drying conditions. At 150 °C, clustering and agglomeration became more pronounced, while the 300× micrographs showed compact irregular particles with folded or collapsed surface features. The 180 °C sample remained highly heterogeneous at 100×, with compact rough surfaces and angular fractured structures at 300×. Overall, the progressive shift toward greater fragmentation, compaction, and agglomeration with increasing drying temperature reflects matrix shrinkage and structural collapse, which Elavarasan and Shamasundar [79] and Gómez-Guillén et al. [15] both attributed to the cumulative effects of thermal energy on the structural integrity of dried protein-rich matrices. The morphological heterogeneity observed across all treatments is also attributable to the multi-tissue composition of whole catfish powder. Gómez-Guillén et al. [15] reported that morphological differences in dried marine protein powders directly influenced their dispersion behavior. This is in accordance with the dispersion characteristics of CFP (Table 9).

### 3.5. Dispersion Characteristics

The dispersion characteristics of CFP were significantly influenced by drying temperature, as reflected by systematic changes in zeta potential, particle size, and polydispersity index (Table 9). Zeta potential became progressively less negative with increasing drying temperature, shifting from −35.82 ± 1.14 mV at 60 °C to −33.47 ± 0.97, −29.83 ± 1.23, −25.61 ± 0.84, and −20.74 ± 1.07 mV at 90, 120, 150, and 180 °C, respectively. As shown in Table 9, this represents a strictly monotonic decrease in absolute zeta potential magnitude across the full temperature range studied, from 35.82 mV at 60 °C to 20.74 mV at 180 °C. This trend indicates that higher drying temperatures weakened the electrostatic stability of the dispersions, as reduced zeta potential magnitude reflects diminished inter-particle repulsive forces and an increased tendency toward aggregation. This shift is likely attributable to heat-induced alterations in the composition, thermal stability, and surface-active functionality of the catfish muscle protein components, including denatured myofibrillar proteins and gelatin derived from collagen in connective tissues, which collectively influenced the emulsifying behavior and physical characteristics of the protein fractions [25]. A corresponding increase in particle size was observed, rising from 312.5 ± 9.4 nm at 60 °C to 328.7 ± 8.6, 368.4 ± 1.2, 418.9 ± 2.7, and 468.3 ± 4.5 nm at 90, 120, 150, and 180 °C, respectively. The progressive enlargement of dispersed particles at higher drying temperatures suggests that more severe thermal treatment promoted stronger intermolecular interactions and reduced the capacity of the powder to redisperse into smaller discrete particles. Given that the powder was produced from cooked whole fish including skin and bone, the dispersed system likely contained a heterogeneous mixture of denatured proteins and collagen-derived materials alongside mineral and lipid-related components. Protein denaturation and collagen structural changes under severe drying conditions may have further promoted molecular aggregation. The PDI increased in parallel, from 0.298 ± 0.011 at 60 °C to 0.315 ± 0.010, 0.342 ± 0.012, 0.378 ± 0.014, and 0.421 ± 0.010 at 90, 120, 150, and 180 °C, indicating that the particle size distribution became progressively broader and less uniform as drying severity increased. The combined increase in particle size and PDI therefore demonstrates that higher drying temperatures not only promoted the formation of larger dispersed particles but also reduced the overall homogeneity of the dispersion.

These changes were directly reflected in the colloidal stability classification, which declined progressively with increasing drying temperature. Samples dried at 60 and 90 °C were classified as stable, the 120 °C sample as moderately stable, the 150 °C sample as approaching unstable, and the 180 °C sample as unstable. This decline is consistent with the reduction in zeta potential magnitude and the simultaneous increases in particle size and PDI, collectively indicating that the colloidal integrity of the dispersions was progressively compromised at higher drying temperatures. Overall, lower drying temperatures were more favorable for maintaining electrostatically stable, relatively small, and homogeneous dispersions, whereas higher drying temperatures progressively impaired colloidal stability and promoted particle aggregation in whole catfish powder dispersions.

### 3.6. Lipid Degradation and Oxidative Stability

Thermal processing substantially influenced the lipid stability of catfish powder, as reflected by changes in free fatty acid content (FFA), peroxide value (PV), and thiobarbituric acid reactive substances (TBARS) across drying temperatures (Table 10). These indicators collectively assess the extent of hydrolytic and oxidative lipid degradation, both of which are central to evaluating the chemical stability and storage quality of dried fish products. FFA content was highest at 90 °C (1.88 ± 1.40%), indicating active lipid hydrolysis under moderate thermal conditions. This behavior is consistent with the preservation of endogenous lipase activity at sub-denaturing temperatures, which catalyzes the hydrolysis of triglycerides into free fatty acids. Similar patterns have been reported in dried fish products where moderate drying temperatures are insufficient to fully inactivate lipolytic enzymes, resulting in elevated hydrolytic activity [80]. As drying temperature increased beyond 90 °C, FFA content declined sharply, reaching its lowest value at 180 °C (0.69 ± 0.02%). This decline reflects progressive thermal inactivation of lipase activity at elevated temperatures, potentially combined with volatilization or secondary degradation of free fatty acids, consistent with findings reported for thermally processed fish [18]. In contrast, PV and TBARS followed the opposite trend, increasing progressively with drying temperature. Although PV showed no significant differences across treatments, it increased numerically from 1.20 ± 0.02 meq/kg at 60 °C to 1.82 ± 0.23 meq/kg at 180 °C. TBARS values similarly rose from 1.19 ± 0.26 mg MDA/kg at 60 °C to 5.36 ± 0.34 mg MDA/kg at 180 °C, indicating accumulation of secondary oxidation products with increasing thermal severity. These results demonstrate that higher drying temperatures accelerated lipid peroxidation, promoting the formation of both primary and secondary oxidative degradation products, in agreement with prior research on lipid oxidation in dried fish systems [16,81]. Among the tested conditions, drying at 120 °C produced the most favorable lipid stability profile. At this temperature, FFA content declined to 1.40 ± 0.12%, indicating substantial suppression of enzymatic hydrolysis, while PV (1.45 ± 0.13 meq/kg) and TBARS (3.11 ± 0.15 mg MDA/kg) remained at intermediate levels, reflecting controlled rather than excessive oxidative degradation. This temperature range has been identified in previous studies as effective for balancing enzymatic inactivation with acceptable levels of lipid oxidation in thermally processed fish products [11,16,81]. Overall, drying at 120 °C effectively suppressed hydrolytic degradation while maintaining oxidative stability within acceptable limits, making it the most favorable condition among those tested for preserving the lipid integrity and chemical stability of whole catfish powder.

### 3.7. Flavor Profile

**Figure 7:**
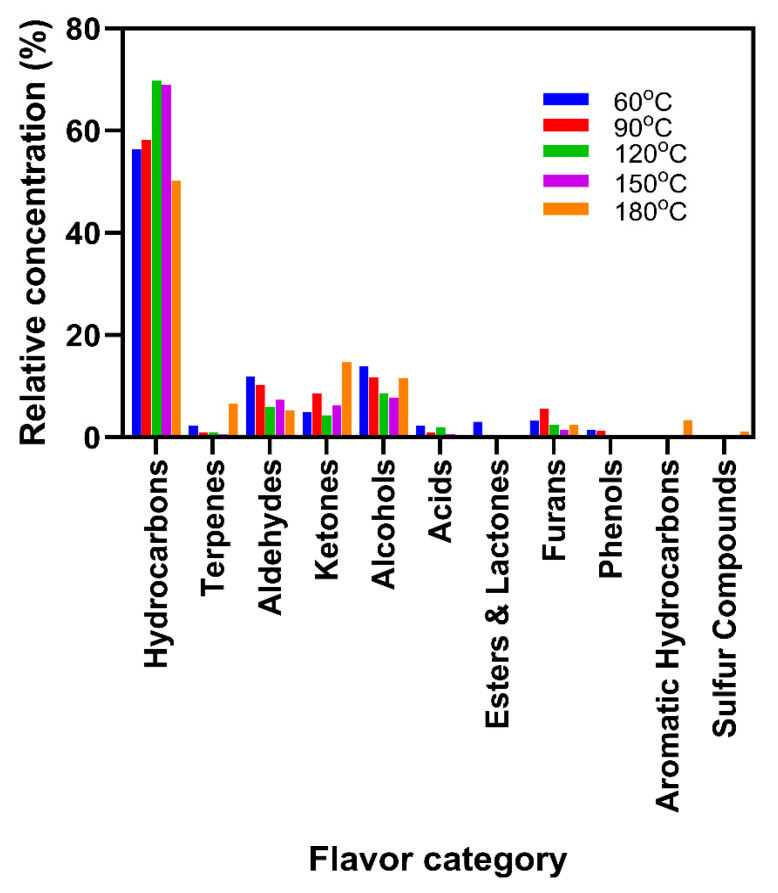
Flavor category of CFP dried at different tray-drying temperatures.

The volatile flavor profile of CFP was significantly affected by the drying temperature, as reflected by systematic shifts in the relative abundance and chemical class distribution of the identified volatile organic compounds (VOCs) (Table 11). Overall, the flavor profile of CFP revealed a total of 72 VOCs across the five drying treatments, grouped into 11 chemical classes, with the cumulative relative content ranging from 93.07 to 99.21%, indicating that the GC–MS profile captured the majority of the volatile fraction in CFP (Figure 7). Across all treatments, hydrocarbons, alcohols, aldehydes, ketones, and furans were the dominant contributors, whereas terpenes, aromatic hydrocarbons, sulfur compounds, esters, lactones, acids, and phenols were comparatively minor and showed strong temperature-dependent emergence or disappearance. Studies have reported that drying temperatures, processing time, and moisture loss play crucial roles in the formation of VOCs, particularly lipid-derived, Maillard, and Strecker-type volatiles, in dried aquatic and meat products [82,83].

Among the VOCs, hydrocarbons constituted the most abundant chemical class in CFP, with relative content ranging from 50.23% (180 °C) to 69.86% (120 °C). The hydrocarbon fraction was higher at 120 °C and 150 °C than at 60 °C, 90 °C, or 180 °C, indicating that intermediate-to-high drying temperatures favored their accumulation. Octane, 3,5-dimethyl- emerged as the dominant individual compound (10.24–28.32%), followed by nonane, 3-methyl-, 2,4-dimethyl-1-heptene, dodecane, and heptane, 2,2,4,6,6-pentamethyl-, all of which are typically associated with the secondary decomposition of fatty acid hydroperoxides and protein side-chain pyrolysis [84]. In contrast, the marked decline in hydrocarbons at 180 °C (50.23%) coincided with the appearance of new chemical classes, including aromatic hydrocarbons (3.36%) and sulfur compounds (1.14%), suggesting that the carbon skeleton previously trapped in saturated alkanes could be redirected into aromatic pathway and Strecker-pathway products at an elevated temperature. A similar redistribution has been documented in hot-air-dried Antarctic krill, where high-temperature treatment shifted the volatile profile from a hydrocarbon-rich profile to a roasted-flavor profile dominated by ketones, aromatic species, and heterocycles [81].

Aldehydes were the second most abundant class in CFP samples dried at 60 °C and 90 °C, contributing 11.85 and 10.21% to the total volatile fraction, respectively. However, this contribution progressively declined to 5.90, 7.38, and 5.21% at 120 °C, 150 °C, and 180 °C, respectively. In general, hexanal, considered the principal autoxidation product of n-6 polyunsaturated fatty acids (mainly linoleic and arachidonic acid), was the most abundant aldehyde and decreased significantly from 7.29 ± 0.36% at 90 °C to 3.44 ± 0.34% at 180 °C (*p* < 0.05). A similar declining trend was observed for nonanal, octanal, and (E)-2-octenal, all of which are well-documented secondary lipid oxidation products derived from the breakdown of oleic and linoleic acid hydroperoxides [85]. In addition, hexanal, nonanal, and octanal are widely reported as the principal contributors to grassy, rancid, and fishy off-odors in dried fish products [86], and a similar volatile signature has been documented previously in farm-raised hybrid catfish muscle stored under refrigeration, where these aldehydes were directly linked to the development of a fishy off-odor [87]. The progressive reduction of these aldehydes at 120 °C and above could be attributed to two parallel processes: (i) faster moisture removal, which shortens the residence time available for hydroperoxide chain propagation, and (ii) the volatilization or further degradation of low-molecular-weight aldehydes once the surface temperature exceeded 100 °C. This is in accordance with the results of lipid oxidation in this study (Table 10), where TBARS values plateaued at higher drying temperatures, despite longer cumulative thermal exposure. Overall, the results showed that drying at 120 °C effectively suppressed the formation of fishy and grassy aldehyde off-flavors without triggering the appearance of undesirable Strecker-type volatiles, an outcome that is highly desirable for downstream food fortification applications [81].

Alcohol was another major class in CFP, ranging from 7.75 to 13.93% across the five treatments, with the 60 °C sample having the highest content. The most prominent compound was 2-butyl-1-octanol, which decreased gradually from 6.62 ± 0.29% at 60 °C to 4.04 ± 0.31% at 180 °C. In contrast, 1-octen-3-ol, a key marker of n-6 PUFA enzymatic and thermal oxidation, often described as having a mushroom-like or earthy odor, exhibited a non-monotonic trend, increasing from 2.71% at 60 °C to 4.41% at 90 °C, then decreasing at 120 °C and 150 °C, and rising sharply to 6.50 ± 0.28% at 180 °C. The observed changes in 1-octen-3-ol can be attributed to residual lipoxygenase activity at low to moderate drying temperatures and the thermal decomposition of linoleic acid hydroperoxides at higher temperatures [85,86]. 1-Octen-3-ol has been previously identified as a discriminating volatile compound in hot-air-dried shrimp, dried eel, and dry-cured fish, where it usually negatively affects consumer acceptability when present above its odor threshold [88]. The lower 1-octen-3-ol levels recorded at 120 and 150 °C provide further support for the suitability of these temperatures for producing CFP with reduced characteristic fishy notes.

Similarly, the ketones increased substantially with drying temperature, from 4.91% at 60 °C to 14.67% at 180 °C, becoming the second most abundant class in the highest-temperature sample. Methyl ketones, such as 2-decanone, 2-nonanone, 2-heptanone, and 3,5-octadien-2-one, were the main contributors, with 2-decanone (5.25 ± 0.24%) detected exclusively at 180 °C. The progressive accumulation of methyl ketones is frequently associated with the β-keto acid decarboxylation pathway, lipid–Maillard interactions, and Strecker degradation of free amino acids in the presence of α-dicarbonyl compounds [53,82]. Furthermore, two additional volatile classes emerged exclusively at 180 °C in the CFP, namely aromatic hydrocarbons (benzyl alcohol and 4-methyl-benzenemethanol; total 3.36%) and sulfur compounds (1.14%), which are characteristic Strecker-type products of phenylalanine and sulfur-containing amino acids (cysteine and methionine), respectively. Furthermore, the terpene class showed a marked increase at 180 °C (6.63%), with (1S)-(–)-camphor accounting for 4.85%, likely released from the matrix or generated through thermally promoted rearrangements of feed-derived terpenoid precursors. A similar progression from a lipid-oxidation-dominated to a Maillard/Strecker-dominated flavor profile has been documented in dry-cured Spanish mackerel and hot-air-dried shrimp, where free amino acids serve as the limiting precursors for pyrazine, ketone, and aromatic product formation [89,90]. The furan class was characterized mainly by 2-pentyl-furan, a recognized linoleic acid autoxidation marker that has been linked to beany, green, and fishy off-notes [86]. Its content peaked at 90 °C (5.01 ± 0.21%) and decreased markedly at 120, 150, and 180 °C (1.46–2.36%). Phenols such as methyleugenol and butylated hydroxytoluene (BHT) steadily declined from 1.42% at 60 °C to undetectable levels at 180 °C, consistent with the thermal lability of these compounds and their well-known volatility under prolonged heating. Esters and lactones were detected only at 60 and 90 °C and disappeared at 120 °C and above, whereas free fatty acids (mainly hexanoic acid) decreased from 2.22% at 60 °C to 0.12% at 180 °C, in line with their volatilization and partial conversion to ketones and esters at higher drying temperatures [84].

The results demonstrated that the volatile profile of CFP was influenced by the drying temperature. At temperatures ranging from 60 to 90 °C, lipid oxidation volatiles such as hexanal, nonanal, 2-pentyl-furan, and 1-octen-3-ol, which are associated with fishy odors, were predominant [85,87]. Conversely, at temperatures between 150 and 180 °C, there was an increase in Maillard/Strecker reaction products, including methyl ketones and sulfur compounds, which correlated with browning. At 120 °C, both pathways were minimized, resulting in reduced levels of fishy aldehydes (5.90%) and 1-octen-3-ol (3.45%), alongside limited accumulation of Maillard-derived methyl ketones and sulfur compounds relative to 150–180 °C. This intermediate volatile profile, characterized by lower relative peak areas of lipid-oxidation markers than at 60–90 °C and lower relative abundance of Maillard and Strecker products than at 150–180 °C, is further supported by the intermediate TBARS values and comparatively better retention of unsaturated fatty acids at 120 °C (Table 6 and Table 10), suggesting that 120 °C produced the most compositionally balanced volatile fraction among the tested treatments. The volatile profile of CFP dried at 120 °C may be preliminarily considered more compatible with neutral or savory food matrices such as instant noodles; however, this inference is based solely on GC-MS compositional data and requires confirmation through odor activity value (OAV) analysis using compound-specific odor thresholds and a trained sensory panel evaluation.

### 3.8. Microbial Safety

Microbial analysis of CFP demonstrated that increasing drying temperature progressively reduced aerobic mesophilic bacterial and fungal contamination, while pathogenic microorganisms remained absent or below the threshold detection limit across all treatments (Table 12), in accordance with the standards established by the Thai Community Product Standard (TCPS 6/2546, TCPS 98/2546, TCPS 134/2546 and TCPS 300/2547) [91]. This temperature-dependent reduction in microbial load reflects the well-established lethal effects of thermal treatment on microbial cell integrity and enzymatic function, whereby higher temperatures induce irreversible structural damage and inhibit the metabolic activities necessary for microbial survival and growth [92]. The observed pattern is consistent with prior findings on dried fish and seafood products, confirming that drying temperature is a primary determinant of microbial stability in fish-based powder systems [11]. At 60 °C, the total plate count (TPC) reached 4.5 × 10^3^ CFU g^−1^ and yeast and mold counts were detected at 1.2 × 10^2^ CFU g^−1^, while *Salmonella*, total coliforms, and *E. coli* remained absent or below 3 MPN g^−1^. The relatively elevated microbial counts at this temperature indicate that mild drying conditions were insufficient to fully inactivate heat-resistant mesophilic bacteria and fungal spores [93]. The residual moisture retained at 60 °C likely provided conditions conducive to limited microbial survival, consistent with reports that inadequate thermal treatment in dried fish results in partial microbial persistence [93]. The absence of pathogenic microorganisms at this stage suggests that hygienic handling of raw materials and processing conditions were adequately maintained. At 90 °C, microbial counts declined markedly, with TPC reduced to 8.5 × 10^2^ CFU g^−1^ and yeast and mold counts falling to 1.5 × 10^1^ CFU g^−1^, while pathogenic indicators remained unchanged. This substantial reduction at moderate temperatures is attributable to the lethal disruption of microbial cell membranes and suppression of metabolic activity under elevated thermal conditions [92]. The combined effect of increased temperature and reduced water activity at this stage likely contributed to the inactivation of the majority of vegetative microorganisms [92]. At 120 °C, TPC declined further to 1.5 × 10^2^ CFU g^−1^ and yeast and mold were no longer detectable, while all pathogenic indicators remained absent or below the detection limit. The complete elimination of fungal counts at this stage is consistent with the known thermal sensitivity of fungal spores, which are generally destroyed at temperatures exceeding 100 °C [94,95]. At 150 and 180 °C, TPC values fell below the detection limit (<10 CFU g^−1^), and yeast and mold, total coliforms, *E. coli*, and *Salmonella* were all undetected, confirming that drying at these temperatures achieved near-complete microbial inactivation and ensured product safety [93]. Overall, microbial data indicate that drying temperature above 120 °C effectively eliminated detectable microbial contamination in whole catfish powder. However, the quality implications of higher drying temperatures, including progressive protein denaturation and lipid oxidation as demonstrated in the preceding analyses, must be considered alongside microbial safety when selecting the optimal drying condition for practical applications [11]. Notably, all CFP treatments attained aw values below 0.60 (range 0.23–0.42; Table 2), a threshold below which the growth of virtually all spoilage bacteria, yeasts, and most molds is effectively inhibited, providing an intrinsic storage hurdle that complements the thermal inactivation achieved during drying.

## 4. Conclusions

Overall, the present study revealed that drying temperature significantly affected the physicochemical, nutritional, functional, structural, volatile flavor, oxidative stability, and microbiological quality of pre-cooked whole catfish powder (*Clarias macrocephalus* × *C. gariepinus*), a product derived from whole fish including bone and skin and subjected to pre-cooking prior to tray drying. No single drying temperature was optimal across all quality criteria simultaneously: drying at 60 °C best preserved functional properties and n-3 polyunsaturated fatty acid retention but was least effective for moisture reduction and microbial inactivation, whereas drying at 150–180 °C achieved the lowest water activity and most effective microbial control at the expense of substantial losses in heat-sensitive essential amino acids, n-3 PUFAs, and functional performance attributes. When all measured quality parameters were considered together, 120 °C provided the most favorable overall balance of dehydration efficiency, nutritional retention, functional performance, oxidative stability, and microbiological safety, and is therefore recommended as the optimal processing condition for catfish powder production. Ash content increased substantially with drying temperature, primarily as a consequence of progressive fat loss-driven compositional redistribution rather than true mineral enrichment, while individual mineral concentrations were only marginally affected by drying temperature, and structural analyses indicated that the major chemical and crystalline characteristics of the powder were largely preserved across all tested conditions, despite moderate molecular, morphological, and thermal modifications that became more pronounced at higher temperatures. A formal drying kinetics analysis, including drying rate curves, Page/Midilli-Kucuk model fitting, and energy efficiency calculations, was beyond the scope of the present study and should be incorporated in future work to fully characterize drying behavior across the tested temperature range and to quantify specific energy input per unit of moisture removed. The catfish powder produced at 120 °C could possibly serve as a functional ingredient in value-added food product development and may be applied in various food formulations to improve the nutritional and quality attributes of the finished product.

## Figures and Tables

**Table 1:** Color characteristics of CFP dried at different tray-drying temperatures.

Sample	L*	a*	b*	C*	h°
CFP 60 °C	49.53 ± 0.41 ^e^	1.94 ± 0.05 ^d^	13.95 ± 0.37 ^c^	14.08 ± 0.38 ^c^	82.07 ± 0.11 ^a^
CFP 90 °C	50.40 ± 0.24 ^d^	2.71 ± 0.08 ^c^	14.14 ± 0.10 ^c^	14.44 ± 0.10 ^c^	80.69 ± 0.08 ^b^
CFP 120 °C	52.79 ± 0.15 ^c^	2.82 ± 0.02 ^b^	15.43 ± 0.24 ^b^	15.68 ± 0.24 ^b^	80.10 ± 0.33 ^c^
CFP 150 °C	53.46 ± 0.53 ^b^	2.92 ± 0.02 ^a^	15.53 ± 0.08 ^b^	15.77 ± 0.07 ^b^	79.64 ± 0.19 ^d^
CFP 180 °C	57.64 ± 0.27 ^a^	2.97 ± 0.01 ^a^	18.14 ± 0.21 ^a^	18.38 ± 0.20 ^a^	78.32 ± 0.02 ^e^

^a,b,c,d,e^ Different superscripts within the same column indicate significant differences (*p* < 0.05). Data represented as mean ± SD (n = 3).

**Table 2:** Process yield, water activity, pH and protein solubility of CFP dried at different tray-drying temperatures.

Sample	Process Yield (%)	Water Activity	pH	Protein Solubility (% N_2_)
CFP 60 °C	11.66 ± 0.18 ^a^	0.42 ± 0.02 ^a^	7.88 ± 0.01 ^a^	4.12 ± 0.09 ^a^
CFP 90 °C	11.41 ± 0.17 ^a^	0.42 ± 0.01 ^a^	7.87 ± 0.02 ^a^	3.96 ± 0.08 ^b^
CFP 120 °C	11.17 ± 0.56 ^ab^	0.36 ± 0.01 ^b^	7.82 ± 0.01 ^b^	3.71 ± 0.07 ^c^
CFP 150 °C	10.91 ± 0.05 ^b^	0.29 ± 0.02 ^c^	7.79 ± 0.01 ^c^	3.42 ± 0.06 ^d^
CFP 180 °C	10.18 ± 0.08 ^c^	0.23 ± 0.02 ^d^	7.73 ± 0.01 ^d^	3.18 ± 0.05 ^e^

^a,b,c,d,e^ Different superscripts within the same column indicate significant differences (*p* < 0.05). Data represented as mean ± SD (n = 3).

**Table 3:** Proximate composition of CFP dried at different tray-drying temperatures.

Sample	Moisture (%)	Fat (%)	Protein (%)	Ash (%)	Carbohydrate (%)
CFP 60 °C	6.24 ± 0.10 ^a^	22.49 ± 0.15 ^a^	57.63 ± 0.03 ^e^	5.28 ± 0.10 ^e^	8.36 ± 0.08 ^a^
CFP 90 °C	5.91 ± 0.01 ^b^	22.36 ± 0.08 ^a^	57.90 ± 0.15 ^d^	8.47 ± 0.64 ^d^	5.36 ± 0.72 ^b^
CFP 120 °C	5.04 ± 0.04 ^c^	21.93 ± 0.04 ^b^	59.23 ± 0.06 ^c^	11.01 ± 0.13 ^c^	2.79 ± 0.18 ^c^
CFP 150 °C	5.03 ± 0.05 ^c^	20.77 ± 0.05 ^c^	60.58 ± 0.05 ^b^	11.74 ± 0.21 ^b^	1.88 ± 0.21 ^d^
CFP 180 °C	4.75 ± 0.03 ^d^	17.17 ± 0.01 ^d^	62.24 ± 0.16 ^a^	15.14 ± 0.13 ^a^	0.70 ± 0.22 ^e^

^a,b,c,d,e^ Different superscripts within the same column indicate significant differences (*p* < 0.05). Data represented as mean ± SD (n = 3).

**Table 4:** Mineral composition of CFP dried at different tray-drying temperatures.

Sample	Mineral Composition (mg/100 g Samples, Dry Basis)							
	Ca	P	K	Na	Mg	Fe	Zn	Mn
CFP 60 °C	748.25 ± 5.43 ^e^	412.35 ± 3.87 ^e^	521.48 ± 4.23 ^e^	198.35 ± 2.14 ^e^	68.42 ± 1.23 ^e^	18.95 ± 0.42 ^a^	5.84 ± 0.15 ^a^	1.42 ± 0.04 ^e^
CFP 90 °C	756.83 ± 4.91 ^d^	418.72 ± 4.15 ^d^	528.63 ± 3.96 ^d^	201.47 ± 1.98 ^d^	69.15 ± 0.98 ^d^	18.72 ± 0.38 ^a^	5.79 ± 0.12 ^a^^b^	1.44 ± 0.03 ^d^
CFP 120 °C	768.47 ± 6.12 ^c^	427.54 ± 3.64 ^c^	538.91 ± 4.57 ^c^	205.83 ± 2.36 ^c^	70.28 ± 1.14 ^c^	18.43 ± 0.45 ^b^	5.71 ± 0.14 ^b^	1.47 ± 0.05 ^c^
CFP 150 °C	779.26 ± 5.28 ^b^	436.18 ± 4.02 ^b^	549.37 ± 3.81 ^b^	210.26 ± 2.07 ^b^	71.47 ± 1.07 ^b^	18.04 ± 0.51 ^c^	5.62 ± 0.11 ^c^	1.49 ± 0.03 ^b^
CFP 180 °C	793.54 ± 6.47 ^a^	448.73 ± 4.38 ^a^	563.82 ± 5.14 ^a^	216.74 ± 2.53 ^a^	72.95 ± 1.32 ^a^	17.61 ± 0.47 ^d^	5.51 ± 0.13 ^d^	1.52 ± 0.04 ^a^

^a,b,c,d,e^ Different superscripts within the same column indicate significant differences (*p* < 0.05). Data represented as mean ± SD (n = 3).

**Table 5:** Amino acid profile of CFP dried at different tray-drying temperatures.

Amino Acid (g/100 g Protein)		CFP 60 °C	CFP 90 °C	CFP 120 °C	CFP 150 °C	CFP 180 °C
**Essential amino acids**	Lysine	6.21 ± 0.09 ^c^	6.54 ± 0.07 ^b^	6.75 ± 0.08 ^a^	6.78 ± 0.06 ^a^	6.71 ± 0.07 ^a^^b^
	Leucine	7.15 ± 0.06 ^a^	7.12 ± 0.05 ^a^	7.08 ± 0.07 ^a^^b^	7.02 ± 0.05 ^b^	6.94 ± 0.08 ^c^
	Isoleucine	4.12 ± 0.05 ^a^	4.09 ± 0.04 ^a^	4.05 ± 0.06 ^a^^b^	4.00 ± 0.04 ^b^	3.94 ± 0.05 ^c^
	Valine	4.78 ± 0.06 ^a^	4.75 ± 0.04 ^a^	4.71 ± 0.05 ^a^^b^	4.65 ± 0.06 ^b^	4.58 ± 0.05 ^b^
	Threonine	4.18 ± 0.04 ^b^	4.22 ± 0.05 ^a^^b^	4.25 ± 0.04 ^a^	4.19 ± 0.05 ^a^^b^	4.08 ± 0.06 ^c^
	Methionine	2.71 ± 0.04 ^a^	2.63 ± 0.03 ^b^	2.52 ± 0.04 ^c^	2.38 ± 0.03 ^d^	2.21 ± 0.04 ^e^
	Phenylalanine	3.91 ± 0.05 ^a^	3.89 ± 0.04 ^a^	3.85 ± 0.05 ^a^^b^	3.80 ± 0.04 ^b^	3.74 ± 0.05 ^c^
	Histidine	2.12 ± 0.03 ^a^	2.10 ± 0.04 ^a^	2.09 ± 0.03 ^a^	2.07 ± 0.05 ^a^	2.04 ± 0.03 ^a^
	Tryptophan	0.81 ± 0.02 ^d^	0.87 ± 0.03 ^c^	0.92 ± 0.02 ^b^	0.95 ± 0.02 ^a^^b^	0.97 ± 0.03 ^a^
	Arginine	5.58 ± 0.07 ^a^	5.55 ± 0.05 ^a^	5.51 ± 0.06 ^a^	5.46 ± 0.04 ^a^^b^	5.39 ± 0.07 ^b^
**Non-essential amino acids**	Glutamic acid	13.52 ± 0.11 ^a^	13.45 ± 0.09 ^a^	13.38 ± 0.10 ^a^	13.28 ± 0.08 ^a^	13.12 ± 0.11 ^b^
	Aspartic acid	9.18 ± 0.08 ^a^	9.12 ± 0.07 ^a^	9.05 ± 0.08 ^a^^b^	8.94 ± 0.06 ^b^	8.78 ± 0.09 ^c^
	Glycine	6.42 ± 0.06 ^c^	6.49 ± 0.05 ^b^^c^	6.57 ± 0.06 ^b^	6.67 ± 0.05 ^a^	6.75 ± 0.07 ^a^
	Alanine	5.85 ± 0.05 ^a^	5.82 ± 0.04 ^a^	5.79 ± 0.05 ^a^	5.74 ± 0.05 ^a^	5.67 ± 0.07 ^b^
	Serine	3.71 ± 0.04 ^a^	3.69 ± 0.03 ^a^	3.65 ± 0.04 ^a^^b^	3.60 ± 0.04 ^b^	3.54 ± 0.03 ^c^
	Proline	3.25 ± 0.05 ^c^	3.31 ± 0.04 ^b^^c^	3.38 ± 0.05 ^b^	3.46 ± 0.04 ^a^^b^	3.54 ± 0.06 ^a^
	Tyrosine	2.91 ± 0.03 ^a^	2.88 ± 0.04 ^a^	2.84 ± 0.03 ^a^^b^	2.79 ± 0.05 ^b^	2.72 ± 0.03 ^c^
	Cysteine	1.28 ± 0.02 ^a^	1.24 ± 0.03 ^b^	1.18 ± 0.02 ^c^	1.11 ± 0.03 ^d^	1.03 ± 0.02 ^e^

^a,b,c,d,e^ Different superscripts within the same column indicate significant differences (*p* < 0.05). Data represented as mean ± SD (n = 3).

**Table 6:** Fatty acid profile of CFP dried at different tray-drying temperatures.

Fatty Acid	Relative Concentration (g/100 g Total Fatty Acids)				
	CFP 60 °C	CFP 90 °C	CFP 120 °C	CFP 150 °C	CFP 180 °C
**SFA**					
Myristic acid (C14:0)	2.51 ± 0.05 ^d^	2.57 ± 0.05 ^cd^	2.59 ± 0.06 ^c^	2.72 ± 0.06 ^b^	2.79 ± 0.07 ^a^
Palmitic acid (C16:0)	25.12 ± 0.23 ^d^	25.78 ± 0.26 ^c^	27.23 ± 0.30 ^c^	28.72 ± 0.34 ^b^	30.55 ± 0.36 ^a^
Stearic acid (C18:0)	7.48 ± 0.11 ^d^	7.65 ± 0.12 ^cd^	7.78 ± 0.12 ^c^	8.12 ± 0.13 ^b^	8.43 ± 0.15 ^a^
Arachidic acid (C20:0)	0.52 ± 0.02 ^c^	0.55 ± 0.02 ^bc^	0.55 ± 0.02 ^b^	0.60 ± 0.03 ^ab^	0.60 ± 0.03 ^a^
**ΣSFA**	35.63 ± 0.41 ^d^	36.55 ± 0.45 ^d^	38.15 ± 0.50 ^c^	40.16 ± 0.56 ^b^	42.37 ± 0.61 ^a^
**MUFA**					
Palmitoleic acid (C16:1n-7)	8.04 ± 0.10 ^a^	8.00 ± 0.10 ^a^	7.98 ± 0.11 ^ab^	7.84 ± 0.11 ^b^	7.80 ± 0.12 ^c^
Oleic acid (C18:1n-9)	28.15 ± 0.25 ^a^	28.10 ± 0.27 ^a^	27.63 ± 0.31 ^b^	27.31 ± 0.33 ^bc^	26.57 ± 0.36 ^d^
Gondoic acid (C20:1n-9)	1.48 ± 0.03 ^a^	1.46 ± 0.03 ^a^	1.46 ± 0.04 ^a^	1.40 ± 0.04 ^b^	1.41 ± 0.05 ^b^
**ΣMUFA**	37.67 ± 0.38 ^a^	37.56 ± 0.40 ^a^	37.07 ± 0.46 ^b^	36.55 ± 0.48 ^bc^	35.78 ± 0.53 ^c^
**PUFA**					
Linoleic acid (C18:2n-6, LA)	15.08 ± 0.17 ^a^	14.88 ± 0.18 ^ab^	14.54 ± 0.19 ^c^	13.90 ± 0.18 ^d^	13.33 ± 0.20 ^e^
α-Linolenic acid (C18:3n-3, ALA)	1.98 ± 0.05 ^a^	1.91 ± 0.06 ^ab^	1.84 ± 0.06 ^bc^	1.68 ± 0.06 ^d^	1.57 ± 0.06 ^e^
Arachidonic acid (C20:4n-6, AA)	3.51 ± 0.08 ^a^	3.38 ± 0.08 ^b^	3.19 ± 0.09 ^c^	2.96 ± 0.09 ^d^	2.67 ± 0.10 ^e^
Eicosapentaenoic acid (C20:5n-3, EPA)	1.52 ± 0.04 ^a^	1.44 ± 0.05 ^b^	1.34 ± 0.05 ^c^	1.22 ± 0.05 ^d^	1.08 ± 0.05 ^e^
Docosahexaenoic acid (C22:6n-3, DHA)	4.61 ± 0.10 ^a^	4.28 ± 0.11 ^b^	3.87 ± 0.12 ^c^	3.53 ± 0.12 ^d^	3.20 ± 0.13 ^e^
**ΣPUFA**	26.70 ± 0.44 ^a^	25.89 ± 0.48 ^b^	24.78 ± 0.51 ^c^	23.29 ± 0.50 ^d^	21.85 ± 0.54 ^e^
**Derived Nutritional Indices**					
Σn-3 PUFA	8.11 ± 0.16 ^a^	7.63 ± 0.16 ^b^	7.05 ± 0.18 ^c^	6.43 ± 0.17 ^d^	5.85 ± 0.18 ^e^
Σn-6 PUFA	18.59 ± 0.23 ^a^	18.26 ± 0.24 ^a^	17.50 ± 0.27 ^b^	16.62 ± 0.25 ^c^	15.48 ± 0.28 ^d^
n-3/n-6 ratio	0.44 ± 0.01 ^a^	0.42 ± 0.01 ^ab^	0.40 ± 0.01 ^bc^	0.38 ± 0.01 ^c^	0.37 ± 0.01 ^d^
PUFA/SFA ratio	0.75 ± 0.01 ^a^	0.71 ± 0.01 ^b^	0.65 ± 0.01 ^c^	0.58 ± 0.01 ^d^	0.52 ± 0.01 ^e^

^a,b,c,d,e^ Different lowercase superscript letters within the same row indicate significant differences among drying temperature treatments (*p* < 0.05). Data represented as mean ± SD (n = 3). DHA, docosahexaenoic acid; EPA, eicosapentaenoic acid; AA, arachidonic acid; LA, linoleic acid; ALA, α-linolenic acid; SFA, saturated fatty acids; MUFA, monounsaturated fatty acids; PUFA, polyunsaturated fatty acids; CFP, catfish powder.

**Table 7:** Water holding capacity, oil holding capacity, emulsion activity index, emulsion stability and viscosity of CFP dried at different tray-drying temperatures.

Sample	WHC (g/g)	OHC (g/g)	EAI (m^2^/g)	ES (%)	Viscosity (cP)
CFP 60 °C	2.13 ± 0.16 ^a^	1.13 ± 0.04 ^a^	52.14 ± 1.23 ^a^	57.14 ± 1.32 ^a^	7.16 ± 0.13 ^a^
CFP 90 °C	2.01 ± 0.11 ^a^	1.12 ± 0.17 ^a^	48.87 ± 0.94 ^b^	55.38 ± 0.87 ^b^	6.87 ± 0.10 ^b^
CFP 120 °C	1.79 ± 0.15 ^b^	1.04 ± 0.10 ^a^^b^	46.32 ± 1.45 ^c^	52.69 ± 0.73 ^c^	6.71 ± 0.08 ^c^
CFP 150 °C	1.74 ± 0.18 ^b^	0.99 ± 0.07 ^a^^b^	42.18 ± 1.67 ^d^	48.23 ± 0.92 ^d^	6.67 ± 0.09 ^d^
CFP 180 °C	1.65 ± 0.10 ^b^	0.90 ± 0.05 ^b^	38.54 ± 2.01 ^e^	42.10 ± 1.07 ^e^	6.32 ± 0.09 ^e^

^a,b,c,d,e^ Different superscripts within the same column indicate significant differences (*p* < 0.05). Data represented as mean ± SD (n = 3).

**Table 8:** Flow, packing and reconstitution properties of CFP dried at different tray-drying temperatures.

Sample	Carr Index (%)	Hausner Ratio	Bulk Density (g/cm^3^) ^ns^	Tapped Density (g/cm^3^) ^ns^	Wettability (s)	Water-Dispersible Fraction (%)
CFP 60 °C	12.00 ± 0.21 ^a^	1.12 ± 0.00 ^a^	0.40 ± 0.01	0.45 ± 0.01	8.50 ± 2.21 ^c^	44.20 ± 1.10 ^a^
CFP 90 °C	8.74 ± 0.15 ^b^	1.09 ± 0.00 ^b^	0.40 ± 0.01	0.44 ± 0.01	10.88 ± 0.63 ^c^	41.85 ± 1.25 ^a^^b^
CFP 120 °C	9.72 ± 2.29 ^a^^b^	1.10 ± 0.02 ^a^^b^	0.40 ± 0.01	0.44 ± 0.00	12.62 ± 2.03 ^b^^c^	37.90 ± 1.42 ^a^^b^
CFP 150 °C	10.14 ± 0.00 ^a^^b^	1.10 ± 0.00 ^a^^b^	0.39 ± 0.00	0.44 ± 0.00	15.54 ± 2.99 ^a^^b^	34.75 ± 1.36 ^b^
CFP 180 °C	10.54 ± 1.27 ^a^^b^	1.11 ± 0.01 ^a^^b^	0.39 ± 0.02	0.43 ± 0.02	16.82 ± 2.38 ^a^	31.60 ± 1.28 ^b^

^a,b,c^ Different superscripts within the same column indicate significant differences (*p* < 0.05). ns = not significant. Data represented as mean ± SD (n = 3).

**Table 9:** Zeta potential, particle size and PDI of CFP dried at different tray-drying temperatures.

Sample	Zeta Potential (mV)	Particle Size (nm)	PDI
CFP 60 °C	−35.82 ± 1.14 ^e^	312.5 ± 9.4 ^e^	0.298 ± 0.011 ^d^
CFP 90 °C	−33.47 ± 0.97 ^d^	328.7 ± 8.6 ^d^	0.315 ± 0.009 ^c^
CFP 120 °C	−29.83 ± 1.23 ^c^	368.4 ± 10.2 ^c^	0.342 ± 0.012 ^b^
CFP 150 °C	−25.61 ± 0.84 ^b^	418.9 ± 12.7 ^b^	0.378 ± 0.014 ^a^
CFP 180 °C	−20.74 ± 1.07 ^a^	468.3 ± 14.5 ^a^	0.421 ± 0.016 ^a^

^a,b,c,d,e^ Different superscripts within the same column indicate significant differences (*p* < 0.05). PDI = Polydispersity Index. Data represented as mean ± SD (n = 3).

**Table 10:** Lipid oxidation of CFP dried at different tray-drying temperatures.

Sample	FFA (%)	PV (meq I_2_/kg Lipid)	TBARS (mg MDA/kg)
CFP 60 °C	1.57 ± 0.13 ^b^	1.20 ± 0.02 ^ns^	1.19 ± 0.26 ^c^
CFP 90 °C	1.88 ± 0.14 ^a^	1.39 ± 0.25 ^ns^	1.20 ± 0.11 ^c^
CFP 120 °C	1.40 ± 0.12 ^b^	1.45 ± 0.13 ^ns^	3.11 ± 0.15 ^b^
CFP 150 °C	0.91 ± 0.06 ^c^	1.49 ± 0.33 ^ns^	3.26 ± 0.16 ^b^
CFP 180 °C	0.69 ± 0.02 ^d^	1.82 ± 0.23 ^ns^	5.36 ± 0.34 ^a^

^a,b,c,d^ Different superscripts within the same column indicate significant differences (*p* < 0.05). ns = not significant. Data represented as mean ± SD (n = 3).

**Table 11:** Flavor profile of CFP dried at different tray-drying temperatures.

No.	Compound Name	CAS No.	RT (Min)	Match (%)	Relative Concentration (%)				
					60 °C	90 °C	120 °C	150 °C	180 °C
**Hydrocarbons**									
1	Heptane, 3-methyl-	589-81-1	1.72	91.6	1.42 ± 0.15 ^b^	2.58 ± 0.13 ^a^	2.70 ± 0.17 ^a^	2.42 ± 0.15 ^a^	1.56 ± 0.18 ^b^
2	Octane	111-65-9	1.82	93.5	4.26 ± 0.36 ^b^	4.27 ± 0.40 ^b^	8.80 ± 0.33 ^a^	8.94 ± 0.44 ^a^	2.66 ± 0.14 ^c^
3	Octane, 4-methyl-	2216-34-4	2.13	89.3	0.48 ± 0.06 ^d^	2.66 ± 0.20 ^a^	2.42 ± 0.12 ^b^	2.85 ± 0.17 ^a^	1.19 ± 0.13 ^c^
4	2,4-Dimethyl-1-heptene	19549-87-2	2.28	93.7	3.77 ± 0.29 ^c^	6.55 ± 0.28 ^b^	6.17 ± 0.34 ^b^	7.92 ± 0.50 ^a^	3.52 ± 0.18 ^c^
5	(3S,6S)-3,6-Dimethyloctane	999064-71-1	2.75	82.1	0.22 ± 0.04 ^c^	0.62 ± 0.09 ^a^	0.27 ± 0.04 ^c^	0.40 ± 0.05 ^b^	0.18 ± 0.04 ^c^
6	Octane, 4-ethyl-	15869-86-0	3.00	88.8	0.58 ± 0.07 ^c^	0.38 ± 0.04 ^d^	0.93 ± 0.09 ^a^	0.39 ± 0.07 ^d^	0.73 ± 0.10 ^b^
7	Heptane, 2,2,4,6,6-pentamethyl-	13475-82-6	3.11	93.5	4.27 ± 0.39 ^b^	3.61 ± 0.31 ^c^	5.56 ± 0.30 ^a^	1.69 ± 0.22 ^d^	5.16 ± 0.25 ^a^
8	Nonane, 3-methyl-	5911-04-6	3.25	94.9	7.66 ± 0.42 ^b^	7.46 ± 0.48 ^b^	9.23 ± 0.52 ^a^	4.85 ± 0.45 ^c^	7.59 ± 0.42 ^b^
9	Octane, 3,5-dimethyl-	15869-93-9	3.83	92.9	22.09 ± 1.32 ^c^	17.86 ± 0.65 ^d^	25.62 ± 1.10 ^b^	28.32 ± 1.28 ^a^	10.24 ± 0.39 ^e^
10	Cyclopropane, nonyl-	74663-85-7	4.69	85.2	nd	nd	nd	3.03 ± 0.19 ^a^	3.35 ± 0.18 ^a^
11	Nonane, 2,5-dimethyl-	17302-27-1	4.94	68.6	0.09 ± 0.01 ^b^	0.17 ± 0.03 ^a^	nd	nd	nd
12	Nonane, 4,5-dimethyl-	17302-23-7	6.18	79.1	1.16 ± 0.11 ^a^	1.00 ± 0.09 ^a^	0.84 ± 0.09 ^b^	0.84 ± 0.09 ^b^	1.21 ± 0.15 ^a^
13	Tridecane, 2,5-dimethyl-	56292-66-1	7.61	73.0	nd	nd	nd	nd	0.58 ± 0.08 ^a^
14	Decane, 4-ethyl-	1636-44-8	7.85	69.0	nd	nd	nd	nd	0.12 ± 0.02 ^a^
15	Nonane, 5-propyl-	998-35-6	8.08	74.0	nd	nd	nd	nd	0.48 ± 0.06 ^a^
16	Cyclononane, 1,1,4,4,7,7-hexamethyl-	149331-19-1	8.54	67.1	nd	nd	nd	nd	0.05 ± 0.01 ^a^
17	Dodecane	112-40-3	10.02	93.7	6.63 ± 0.27 ^b^	9.45 ± 0.46 ^a^	6.40 ± 0.45 ^b^	5.64 ± 0.32 ^c^	6.95 ± 0.38 ^b^
18	Undecane, 3-methylene-	71138-64-2	11.33	75.1	0.15 ± 0.03 ^b^	nd	nd	0.40 ± 0.07 ^a^	0.40 ± 0.07 ^a^
19	Decane, 5-ethyl-5-methyl-	17312-74-2	11.97	77.8	0.78 ± 0.08 ^a^	nd	nd	nd	nd
20	Pentadecane	629-62-9	12.00	82.2	1.12 ± 0.08 ^a^	0.56 ± 0.06 ^b^	0.50 ± 0.05 ^b^	nd	nd
21	Tetradecane, 2,6,10-trimethyl-	14905-56-7	12.83	71.9	nd	nd	nd	nd	0.24 ± 0.03 ^a^
22	Eicosane	112-95-8	14.23	78.1	0.58 ± 0.09 ^a^	nd	nd	nd	nd
23	Hexadecane, 2,6,10,14-tetramethyl-	638-36-8	14.24	83.8	nd	nd	nd	nd	0.84 ± 0.13 ^a^
24	Nonadecane, 9-methyl-	13287-24-6	16.62	83.9	0.53 ± 0.06 ^a^	nd	nd	nd	nd
25	Tridecane, 3-methyl-	6418-41-3	16.65	85.8	nd	1.00 ± 0.11 ^a^	0.42 ± 0.06 ^c^	0.40 ± 0.08 ^c^	0.77 ± 0.09 ^b^
26	Tetradecane	629-59-4	17.52	79.0	nd	nd	nd	0.92 ± 0.10 ^b^	1.91 ± 0.16 ^a^
27	Tridecane, 3-methylene-	19780-34-8	18.29	65.7	nd	nd	nd	nd	0.21 ± 0.03 ^a^
28	n-Nonylcyclohexane	2883-02-5	21.24	66.9	nd	nd	nd	nd	0.29 ± 0.04 ^a^
29	Cyclopentene, 5-hexyl-3,3-dimethyl-	61142-66-3	24.11	77.0	0.51 ± 0.07 ^a^	nd	nd	nd	nd
**Terpenes**									
30	D-Limonene	5989-27-5	9.39	82.4	0.38 ± 0.07 ^a^	nd	nd	nd	nd
31	(-)-Limonene	999052-94-4	9.42	88.3	nd	1.04 ± 0.10 ^a^	1.00 ± 0.08 ^a^	0.58 ± 0.09 ^b^	0.87 ± 0.11 ^a^
32	3,7,11-Trimethyl-1-dodecyn-3-ol	1604-35-9	19.15	73.2	nd	nd	nd	nd	0.49 ± 0.06 ^a^
33	(1S)-(-)-Camphor	464-48-2	19.41	69.4	nd	nd	nd	nd	4.85 ± 0.25 ^a^
34	1,1,4,7-Tetramethyldecahydro-1H-cyclopropa[e]azulen-4-ol	88728-58-9	20.43	82.9	nd	nd	nd	nd	0.42 ± 0.05 ^a^
35	Bicyclo [5.2.0]nonane, 2-methylene-4,8,8-trimethyl-4-vinyl-	242794-76-9	20.93	89.0	1.91 ± 0.21 ^a^	nd	nd	nd	nd
**Aldehydes**									
36	Hexanal	66-25-1	5.67	92.5	6.11 ± 0.38 ^b^	7.29 ± 0.36 ^a^	4.16 ± 0.22 ^c^	3.60 ± 0.25 ^c^	3.44 ± 0.34 ^d^
37	Octanal	124-13-0	13.36	87.3	0.81 ± 0.10 ^a^	0.34 ± 0.07 ^b^	0.22 ± 0.04 ^b^	0.23 ± 0.03 ^b^	0.34 ± 0.06 ^b^
38	Nonanal	124-19-6	17.09	92.9	3.50 ± 0.29 ^a^	2.26 ± 0.17 ^b^	1.52 ± 0.13 ^c^	3.55 ± 0.29 ^a^	1.17 ± 0.09 ^c^
39	2-Octenal, (E)-	2548-87-0	18.03	76.3	0.30 ± 0.04 ^a^	0.32 ± 0.05 ^a^	nd	nd	nd
40	Cyclohexanebutanal, 2-methyl-3-oxo-, cis-	92485-93-3	18.08	71.0	nd	nd	nd	nd	0.26 ± 0.05 ^a^
41	Benzaldehyde	100-52-7	19.89	72.6	1.13 ± 0.14 ^a^	nd	nd	nd	nd
**Ketones**									
42	2-Heptanone	110-43-0	9.01	76.0	2.22 ± 0.14 ^b^	2.75 ± 0.21 ^a^	0.75 ± 0.08 ^d^	2.43 ± 0.23 ^a^	1.79 ± 0.22 ^c^
43	2-Octanone	111-13-7	13.22	79.9	nd	0.43 ± 0.06 ^a^	0.19 ± 0.03 ^b^	0.23 ± 0.04 ^b^	0.47 ± 0.06 ^a^
44	3-Octanone, 2-methyl-	923-28-4	15.06	81.4	1.46 ± 0.17 ^a^	1.39 ± 0.14 ^a^	1.01 ± 0.12 ^b^	1.19 ± 0.12 ^a^	nd
45	2-Nonanone	821-55-6	16.97	83.0	0.39 ± 0.04 ^d^	1.40 ± 0.13 ^b^	0.56 ± 0.05 ^d^	0.97 ± 0.15 ^c^	3.60 ± 0.34 ^a^
46	3-Octen-2-one, (E)-	18402-82-9	17.45	91.4	nd	nd	nd	nd	1.14 ± 0.14 ^a^
47	2-Decanone	693-54-9	19.36	87.7	nd	nd	nd	nd	5.25 ± 0.24 ^a^
48	3,5-Octadien-2-one	38284-27-4	20.77	70.1	0.45 ± 0.05 ^c^	0.87 ± 0.13 ^b^	0.35 ± 0.07 ^c^	0.39 ± 0.05 ^c^	1.39 ± 0.14 ^a^
49	1,3-Cyclopentanedione, 2-methyl-	765-69-5	23.17	77.5	0.39 ± 0.05 ^d^	1.76 ± 0.20 ^a^	1.39 ± 0.16 ^b^	1.00 ± 0.08 ^c^	0.93 ± 0.11 ^c^
50	5,9,9-Trimethyl-spiro [3.5]non-5-en-1-one	999158-58-3	24.07	72.1	nd	nd	nd	nd	0.10 ± 0.02 ^a^
**Alcohols**									
51	1-Octanol, 2-butyl-	3913-02-8	4.58	86.5	6.62 ± 0.29 ^a^	4.95 ± 0.46 ^b^	4.66 ± 0.33 ^b^	4.54 ± 0.28 ^b^	4.04 ± 0.31 ^c^
52	1-Decanol	112-30-1	4.69	86.0	2.77 ± 0.24 ^a^	nd	nd	nd	nd
53	1-Octanol, 2,2-dimethyl-	2370-14-1	5.42	66.1	0.54 ± 0.06 ^a^	nd	nd	nd	nd
54	2-Methyltetradecan-1-ol	999349-12-6	11.64	83.5	0.40 ± 0.06 ^b^	0.73 ± 0.12 ^a^	nd	nd	nd
55	1-Octen-3-ol	3391-86-4	18.49	92.1	2.71 ± 0.22 ^d^	4.41 ± 0.32 ^b^	3.45 ± 0.26 ^c^	2.93 ± 0.16 ^d^	6.50 ± 0.28 ^a^
56	1-Octanol	111-87-5	20.59	84.8	0.76 ± 0.09 ^c^	1.64 ± 0.17 ^a^	0.53 ± 0.06 ^d^	0.28 ± 0.04 ^e^	1.07 ± 0.08 ^b^
57	Cyclooctyl alcohol	696-71-9	21.41	70.9	0.13 ± 0.02 ^a^	nd	nd	nd	nd
**Acids**									
58	Hexanoic acid	142-62-1	24.30	92.7	1.73 ± 0.14 ^a^	0.92 ± 0.14 ^b^	1.97 ± 0.24 ^a^	0.62 ± 0.06 ^c^	0.12 ± 0.02 ^d^
59	Caprylic acid	999067-84-4	26.69	79.6	0.18 ± 0.03 ^a^	nd	nd	nd	nd
60	Octanoic acid	124-07-2	26.70	78.6	0.31 ± 0.04 ^a^	0.11 ± 0.01 ^b^	nd	nd	nd
**Esters & Lactones**									
61	Carbonic acid, decyl 2-ethylhexyl ester	999707-28-6	5.06	82.2	0.45 ± 0.09 ^a^	0.47 ± 0.08 ^a^	nd	nd	nd
62	Carbonic acid, eicosyl vinyl ester	999892-52-1	17.50	79.2	1.89 ± 0.19 ^a^	nd	nd	nd	nd
63	2-Butenoic acid, 2-methyl-, 2-methylpropyl ester, (E)-	61692-84-0	21.75	78.8	0.70 ± 0.10 ^a^	nd	nd	nd	nd
**Furans**									
64	Furan, 2-pentyl-	3777-69-3	10.87	94.0	2.60 ± 0.23 ^b^	5.01 ± 0.21 ^a^	2.05 ± 0.11 ^c^	1.46 ± 0.14 ^d^	2.36 ± 0.17 ^b^
65	2(3H)-Furanone, 5-ethyldihydro-	695-06-7	22.61	66.1	0.14 ± 0.02 ^b^	0.36 ± 0.06 ^a^	0.37 ± 0.06 ^a^	0.10 ± 0.02 ^b^	nd
66	2-n-Heptylfuran	3777-71-7	24.14	70.3	0.51 ± 0.05 ^a^	0.24 ± 0.04 ^b^	nd	nd	nd
**Phenols**									
67	Butylated Hydroxytoluene (BHT)	128-37-0	25.12	82.0	0.21 ± 0.03 ^a^	0.17 ± 0.03 ^a^	0.09 ± 0.01 ^b^	0.02 ± 0.01 ^c^	nd
68	Phenol	108-95-2	26.15	72.3	0.23 ± 0.03 ^a^	nd	nd	nd	nd
69	Methyleugenol	93-15-2	26.28	96.3	0.98 ± 0.12 ^b^	1.14 ± 0.10 ^a^	0.16 ± 0.02 ^c^	0.04 ± 0.01 ^c^	nd
**Aromatic Hydrocarbons**									
70	Benzyl alcohol	100-51-6	24.82	80.8	nd	nd	nd	nd	0.14 ± 0.02 ^a^
71	Benzenemethanol, 4-methyl-	589-18-4	25.81	93.4	nd	nd	nd	nd	3.22 ± 0.30 ^a^
**Sulfur Compounds**									
72	Sulfurous acid, decyl 2-ethylhexyl ester	999781-43-5	5.13	79.2	nd	nd	nd	nd	1.14 ± 0.12 ^a^

RT = retention time; Match (%) = NIST/Wiley library match factor; nd = not detected; Values are mean ± SD of triplicate analyses (n = 3). Compounds are listed in order of elution within each chemical class. Relative content (%) was calculated as the ratio of individual peak area to total peak area of all identified compounds per sample. ^a,b,c,d^ Different superscripts within the same row indicate significant differences (*p* < 0.05).

**Table 12:** Microbial characteristics of CFP dried at different tray-drying temperatures ^a^.

Drying Temperature	Total Plate Count (CFU/g)	Yeast & Mold (CFU/g)	Total Coliforms (MPN/g)	*E. coli* (in 25 g)	*Salmonella* spp. (in 25 g)
CFP 60 °C	4.5 × 10^3^	1.2 × 10^2^	<3	nd	nd
CFP 90 °C	8.5 × 10^2^	1.5 × 10^1^	<3	nd	nd
CFP 120 °C	1.5 × 10^2^	nd	<3	nd	nd
CFP 150 °C	<10	nd	<3	nd	nd
CFP 180 °C	<10	nd	<3	nd	nd
Standard Limit ^a^	<1 × 10^4^	<100	<3	nd	nd

Note: nd = not detected. Statistical grouping letters were not applied to TPC and yeast and mold due to the presence of below-detection-limit values (<10 CFU/g and nd) within the dataset, which preclude parametric comparison across all treatments. Qualitative parameters (*E. coli*, *Salmonella*) and MPN threshold values (total coliforms) are not amenable to SD reporting or statistical comparison. ^a^ Standard limits adapted from the Thai Community Product Standard (TCPS 6/2546, TCPS 98/2546, TCPS 134/2546 and TCPS 300/2547).

## Data Availability

The original contributions presented in this study are included within the article/Supplementary Materials. Further inquiries can be directed to the corresponding author.

## Supplementary Materials

The following supporting information can be downloaded at: https://www.mdpi.com/article/10.3390/foods15142443/s1. Table S1: Published proximate composition (%, wet weight basis) of fresh *C. gariepinus* and selected dried catfish and freshwater fish powder products.

## Abbreviations

The following abbreviations are used in this manuscript:

AA	Arachidonic acid
ANOVA	Analysis of variance
BHT	Butylated hydroxytoluene
BPW	Buffered peptone water
Ca	Calcium
CFP	Catfish powder
CI	Carr index
Cu	Copper
DG18	di-chloran glycerol agar
DHA	Docosahexaenoic acid
DLS	Dynamic light scattering
DMRT	Duncan’s new multiple range test
DTG	Derivative thermogravimetric
EAI	Emulsifying activity index
EPA	Eicosapentaenoic acid
ESI	Emulsion stability index
FAMEs	Fatty acid methyl esters
Fe	Iron
FTIR	Fourier transform infrared spectroscopy
GC–MS	Gas Chromatography–Mass Spectrometry
HCl	Hydrochloric acid
HE	Hektoen enteric agar
HNO_3_	Nitric acid
HR	Hausner ratio
ICP-OES	Inductively coupled plasma optical emission spectrometry
K	Potassium
KBr	Potassium bromide
KI	Potassium iodide
KOH	Potassium hydroxide
LiOH	Lithium hydroxide
LOD	Lower limit of detection
LST	Lauryl sulfate tryptose
MDA	Malondialdehyde
Mg	Magnesium
Mn	Manganese
MPN	Most probable number
Na	Sodium
Na_2_HPO_4_	Sodium phosphate dibasic
Na_2_S_2_O_3_	Sodium thiosulfate
NaCl	Sodium chloride
NaH_2_PO_4_	Sodium phosphate monobasic
NaOH	Sodium hydroxide
ND	Not detected
OHC	Oil holding capacity
P	Phosphorus
PDI	Polydispersity index
PUFAs	Polyunsaturated fatty acids
PV	Peroxide value
RV	Rappaport–Vassiliadis medium
SD	Standard deviation
SDS	Sodium dodecyl sulfate
SEM	Scanning Electron Microscopy
TBA	2-thiobarbituric acid
TBARS	Thiobarbituric acid reactive substances
TCA	Trichloroacetic acid
TD	Tray drying
TGA	Thermogravimetric analysis
TPC	Total plate count
TT	Tetrathionate broth
VOC	Volatile organic compound
WDI	Water-dispersibility index
WHC	Water holding capacity
XLD	Xylose lysine deoxycholate agar
XRD	X-ray diffraction
Zn	Zinc
ζ-potential	Zeta potential
ρb	Bulk density
ρt	Tapped density
