# Supplementary material for: Effect of Hot-Air Drying Temperature on the Physicochemical, Functional, Compositional, and Structural Properties of Catfish (Clarias macrocephalus × C. gariepinus) Powder
Source: Foods. 2026 Jul 9;15(14):2443. doi: 10.3390/foods15142443 (PMC13408515; doi:10.3390/foods15142443)
Supplement: Supplementary file 1 [file foods-15-02443-s001.zip › foods-4372672-supplementary.pdf]

Table S1. Published proximate composition (%, wet weight basis) of fresh C. gariepinus and selected dried catfish and freshwater fish powder products.

Species	Processing	Proximate composition (%)				
		Moisture	Crude protein	Crude fat	Ash	Reference
C. gariepinus	Fresh	71.30±0.15	19.03±0.46	8.10±0.09	1.05±0.14	[5]
C. gariepinus	Electric oven-dried (60 °C)	3.57±0.03	63.23 ±0.01	13.55±0.02	5.14±0.04	[95]
C. gariepinus	Oven-dried	4.34±0.02	72.84±0.02	16.82±0.01	5.44±0.02	[6]
H. siamensis	Hot-air dried (50-80°C)	7.0-9.7	38.9-42.0	29.7-35.2	9.9-10.6	[7]

Note: Values are means ± standard deviation where reported; *H. siamensis* values represent the range across drying temperatures (50–80°C).

References

5. Abdel-Mobdy, H.E.; Abdel-Aal, H.A.; Souzan, S.L.; Nassar, A.G. Nutritional value of African catfish (*Clarias gariepinus*) meat. *Asian J. Appl. Chem. Res.* **2021**, *8*, 31–39. https://doi.org/10.9734/AJACR/2021/v8i230190.

6. Salami, S.R.; Awoniyi, O.O.; Oladipupo, T.M. Comparative study of quality deterioration and microbiological safety of oven-dried and smoked products from African catfish (*Clarias gariepinus*) at various storage condition. *Int. J. Fish. Aquat. Stud*. **2024**, *12*, 110–116. https://doi.org/10.22271/fish.2024.v12.i1b.2898.

7. Sroy, S.; Avallone, S.; Servent, A.; In, S.; Arnaud, E. Does drying preserve the nutritional quality of small freshwater fish without excessive concentrations of heavy metals? *Curr. Res. Food Sci*. **2023**, *6*, 100489. https://doi.org/10.1016/j.crfs.2023.100489.

95. Aremu, M.O.; Namo, S.B.; Salau, R.B.; Agbo, C.O.; Ibrahim, H. Smoking methods and their effects on nutritional value of African Catfish (*Clarias gariepinus*). *Open Nutraceuticals J.* **2013**, *6*, 105–112. https://doi.org/10.2174/1876396020130830003.
